# An integrative multi-omics analysis based on liquid–liquid phase separation delineates distinct subtypes of lower-grade glioma and identifies a prognostic signature

**DOI:** 10.1186/s12967-022-03266-1

**Published:** 2022-01-29

**Authors:** Jianglin Zheng, Zhipeng Wu, Yue Qiu, Xuan Wang, Xiaobing Jiang

**Affiliations:** 1 Department of Neurosurgery, Union Hospital, Tongji Medical College, Huazhong University of Science and Technology, Wuhan, Hubei China; 2 Department of Otolaryngology, Union Hospital, Tongji Medical College, Huazhong University of Science and Technology, Wuhan, Hubei China

**Keywords:** Liquid–liquid phase separation, Lower-grade glioma, Genomic alterations, Tumor immune microenvironment, Immunotherapy

## Abstract

**Background:**

Emerging evidences have indicated that the aberrant liquid–liquid phase separation (LLPS) leads to the dysfunction of biomolecular condensates, thereby contributing to the tumorigenesis and progression. Nevertheless, it remains unclear whether or how the LLPS of specific molecules affects the prognosis and tumor immune microenvironment (TIME) of patients with lower-grade glioma (LGG).

**Methods:**

We integrated the transcriptome information of 3585 LLPS-related genes to comprehensively evaluate the LLPS patterns of 423 patients with LGG in The Cancer Genome Atlas (TCGA) cohort. Then, we systematically demonstrated the differences among four LLPS subtypes based on multi-omics analyses. In addition, we constructed the LLPS-related prognostic risk score (LPRS) for individualized integrative assessment.

**Results:**

Based on the expression profiles of 85 scaffolds, 355 regulators, and 3145 clients in LGG, we identified four LLPS subtypes, namely LS1, LS2, LS3 and LS4.

We confirmed that there were significant differences in prognosis, clinicopathological features, cancer hallmarks, genomic alterations, TIME patterns and immunotherapeutic responses among four LLPS subtypes. In addition, a prognostic signature called LPRS was constructed for individualized integrative assessment. LPRS exhibited a robust predictive capacity for prognosis of LGG patients in multiple cohorts. Moreover, LPRS was found to be correlated with clinicopathological features, cancer hallmarks, genomic alterations and TIME patterns of LGG patients. The predictive power of LPRS in response to immune checkpoint inhibitor (ICI) therapy was also prominent.

**Conclusions:**

This study provided a novel classification of LGG patients based on LLPS. The constructed LPRS might facilitate individualized prognosis prediction and better immunotherapy options for LGG patients.

**Supplementary Information:**

The online version contains supplementary material available at 10.1186/s12967-022-03266-1.

## Introduction

Currently, the understanding of the pathogenesis of human tumors is incomplete, which dramatically limits the development of effective treatment strategies. Classic perspective is that hallmark characteristics of tumors are acquired through gene-level alterations that disrupt the ‘lock-and-key’ binding type between crucial proteins. However, emerging evidences indicate that a large proportion of tumor malignant phenotypes originate from the intrinsically disordered domains (IDRs) of protein [1–3]. Notably, the function of proteins with IDRs has been proved to be regulated by the liquid–liquid phase separation (LLPS) process [4–6]. LLPS refers to the phenomenon that biological macromolecules (protein or nucleic acid) form a droplet like condensate without surrounding membranes through weak polyvalent interactions [7–9]. LLPS are the formation mechanism of many membraneless organelles, such as stress granule, processing body (P-body) and nuclear speckle. Through forming an independent membraneless compartment by assembling biological macromolecules with a specific function into a specific space, cells could efficiently perform their biological functions, including reshaping chromatin structure, regulating gene transcription and translation, et al. [10, 11]. LLPS is a dynamic process involving the scaffolds, regulators and clients. Scaffolds appear to be essential for the structural integrity of biomolecular condensates. Regulators ensure that biomolecular condensates function properly. Clients reside in condensates only under certain conditions, and often contain components that specifically bind to components in the scaffolds [12]. It has already been confirmed that the LLPS status of many important proteins, including RNA-binding proteins and transcription factors, affect their own biological activities and the regulation of downstream signal pathways^13^, ^14^. For example, YTHDC1 is an RNA-binding protein, and the biomolecular condensate of YTHDC1 formed by LLPS is indispensable for protecting target mRNAs from degradation [15]. A growing understanding of the underlying molecular principles of LLPS has created awareness of their diverse functions in various cellular processes, including the stress response, the regulation of gene expression, and the control of signal transduction [8]. Aberrant phase separation of key molecules may result in disturbance of cellular signal pathways, and lead to a further pathology status of individual. Accumulating studies have revealed that the LLPS process is nonnegligible for the development and treatment of human diseases, including tumors [16, 17]. For instance, the deubiquitylase USP42 leads to nuclear speckle mRNA splicing through dynamic LLPS process to promote tumorigenesis [18]. The LLPS of YAP promoted by promoted by interferon-γ induces cancer resistance to anti-PD-1 immunotherapy [19]. Hence, we consider that exploring the role of LLPS should be a fruitful area in oncology research, and will further benefit the understanding of tumor pathogenesis, the prediction of prognosis, and the individualized selection of treatment options.

Diffuse lower-grade glioma (LGG) is the most common primary central nervous system tumor, characterized by high recurrence and progression rates even with the continuing development of multiple treatment modalities [20]. The marked heterogeneities in prognosis and therapeutic response of patients are always major clinical challenges. In the above context, this study attempted to identify and quantify such heterogeneities based on the LLPS patterns of LGG patients. Finally, four LLPS subtypes of LGG patients in The Cancer Genome Atlas (TCGA) cohort were identified with distinct prognosis, clinicopathological features, hallmark characteristics, genomic alterations, tumor immune microenvironment (TIME) patterns and immunotherapeutic responses. The constructed prognostic signature, namely LLPS-related prognostic risk score (LPRS), exhibited robust predictive power in the prognosis and the response to immune checkpoint inhibitor (ICI) therapy. To our knowledge, the present study is the first to reveal the multi-dimensional heterogeneities of LGG patients based on LLPS. Our findings might facilitate individualized prognosis prediction and better immunotherapy options for LGG patients.

## Methods

### The overall flow diagram

**Fig. 1:**
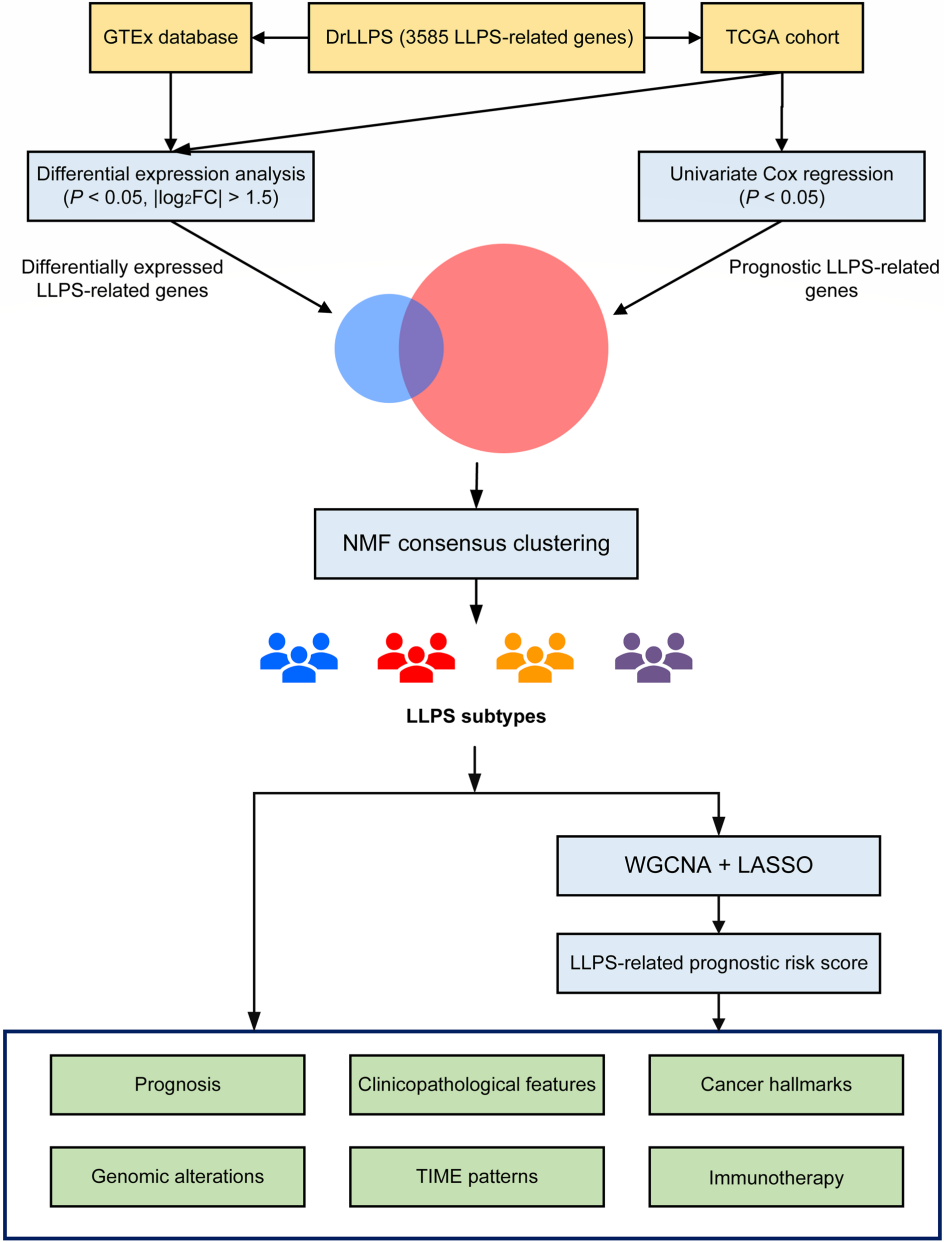
The overall flow diagram of this study

The overall flow diagram of this study was presented in Fig. 1. Firstly, we attempted to screen out the LLPS-related genes with prognostic significance, as well as differential expressions between normal tissues and glioma tissues. Based on the expression profiles of theses selected genes, non-negative matrix factorization (NMF) consensus clustering was performed to construct LLPS subtypes of LGG patients. Subsequently, we explored the multi-dimensional heterogeneities of LLPS subtypes. In addition, the weighted gene co-expression network analysis (WGCNA) and least absolute shrinkage and selection operator (LASSO) Cox algorithm were combined to screen for robust LPRS. The effectiveness of LPRS was assessed in multiple dimensions.

### Patient population and data resource of LLPS

The RNA sequencing (RNA-seq) data and clinical information of LGG patients were extracted from TCGA (https://portal.gdc.cancer.gov/), the Chinese Glioma Genome Atlas (CGGA; http://www.cgga.org.cn/) and Rembrandt (http://gliovis.bioinfo.cnio.es/) databases. Patients with missing survival data or overall survival (OS) < 30 days, or without clear histopathological diagnosis were excluded from further analyses. In total, five cohorts were gathered, including TCGA, CGGA-mRNAseq_693, CGGA-mRNAseq_325, CGGA-mRNA-arry_301 and Rembrandt cohorts. We also downloaded the RNA-seq data of 1152 normal brain tissues from the Genotype-Tissue Expression (GTEx; https://gtexportal.org/home/) database. All RNA-seq data were obtained in the format of fragments per kilobase of exon model per million mapped reads (FPKM) normalized. The clinicopathological features of LGG patients in five cohorts were summarized in Table 1.

**Table 1:** Demographics and clinicopathological features of LGG patients in all cohorts

	TCGA	CGGA-mRNAseq_693	CGGA-mRNAseq_325	CGGA-mRNA-arry_301	Rembrandt
Number of samples	423	420	170	158	119
Age (mean ± SD; years)	43.28 ± 13.34	40.32 ± 10.36	40.39 ± 10.85	39.58 ± 10.57	NA
Gender					
Male	234	235	105	90	59
Female	189	185	65	68	37
NA	0	0	0	0	23
Survival status					
Alive	317	223	82	85	34
Dead	106	197	88	73	85
Pretreatment KPS					
< 80	33	NA	NA	NA	NA
≥ 80	224	NA	NA	NA	NA
NA	166	NA	NA	NA	NA
Histology					
Astrocytoma	154	254	110	102	80
Oligodendroglioma	110	137	60	38	34
Oligoastrocytoma	159	29	0	18	0
NA	0	0	0	0	5
WHO grade					
II	201	172	97	105	63
III	222	248	73	53	56
IDH status					
Mutant	344	288	125	104	NA
Wild type	77	94	44	1	NA
NA	2	38	1	53	NA
1p19q status					
Codeletion	141	125	55	16	8
Non-codeletion	282	257	113	33	13
NA	0	38	2	109	98
MGMT promoter status					
Methylated	351	200	84	43	NA
Unmethylated	72	129	70	106	NA
NA	0	91	16	9	NA
TERT status					
Mutant	120	NA	NA	NA	NA
Wild type	140	NA	NA	NA	NA
NA	163	NA	NA	NA	NA

*SD* standard deviation, *KPS* Karnofsky Performance Score

The data resource of LLPS (DrLLPS; http://llps.biocuckoo.cn/) is an integrative database for proteins involved in LLPS, which has incorporated 150 scaffolds that are drivers of LLPS, 987 regulators that contribute in modulating LLPS, and 8148 clients, all of which were experimentally identified in multiple eukaryotic species [21]. Our study subjects were homo sapiens. Then, a total of 3600 LLPS-related genes were retained after excluding these LLPS-related genes experimentally identified in other eukaryotic species. Finally, a total of 3585 LLPS-related genes (85 scaffolds, 355 regulators and 3145 clients) had available gene-expression data in TGCA cohort and were screened out for subsequent analyses.

### Identification of LLPS subtypes of LGG patients

The expression data of these LLPS-related genes was normalized with log_2_(FPKM + 1) transformation for the differential expression analysis between LGG tissues and normal brain tissues. Then, the differentially expressed genes (DEGs; *P* < 0.05, |log_2_FC|> 1.5) were retained for univariate Cox regression analyses to identify the prognostic LLPS-related DEGs. The Gene Ontology (GO) and Kyoto Encyclopedia of Genes and Genomes (KEGG) pathway analyses were conducted for the functional annotation via the “clusterProfiler” package of R. Based on the expression profiles of prognostic LLPS-related DEGs, the non-negative matrix factorization (NMF) consensus clustering was performed through the R package “NMF” to obtain LLPS subtypes of LGG patients. The cophenetic, dispersion and silhouette indicators were used to determine the optimal clustering number. We applied the t-distributed stochastic neighbor embedding (tSNE) algorithm to confirm the reliability of clustering results by visual inspection. The Kaplan–Meier (K-M) survival curves were used to determine the survival difference among different LLPS subtypes. On the basis of 50 hallmark gene sets retrieved from Molecular Signatures Database (MSigDB), the enrichment levels of 50 hallmarks of different LLPS subtypes were quantified by single-sample Gene Set Enrichment Analysis (ssGSEA).

### Analyses of genomic alterations

The somatic mutation profiles sorted in the form of mutation annotation format (MAF) were obtained from TCGA database. By using the R package “Maftools”, we analyzed and visualized the mutation profiles and frequencies of genes in different LLPS subtypes. The tumor mutation burden (TMB) was calculated as mutations per megabase (mut/Mb) [22]. Also, the copy number alteration (CNA) data of LGG patients were acquired from TCGA database. We used GISTIC2.0 to identify significant amplifications or deletions in the whole genome [23]. The CNA burden was defined as the total number of genes with copy number changes at the focal and arm levels [24].

### Assessment of TIME and immunotherapeutic responses

ESTIMATE algorithm was employed to calculate the immune scores, stromal scores, ESTIMATE scores and tumor purity of LGG patients via the R package “estimate” [25]. The enrichment scores of 29 immune signatures were quantified by ssGSEA [26]. Based on the ssGSEA Z-scores of those 29 immune signatures, patients were classified into high-/low- immunity subtypes. We also applied the CIBERSORT algorithm with 1,000 permutations to calculate the compositions of 22 types of immune cells [27]. The Tumor Immune Dysfunction and Exclusion (TIDE) algorithm was performed online (http://tide.dfci.harvard.edu/) to assess the potential response to ICI therapy. Patients with lower TIDE scores or higher microsatellite instability (MSI) scores were more likely to show stronger responses to ICI therapy. Another method, unsupervised subclass mapping (https://cloud.genepattern.org/gp), was also utilized to predict the response to ICI therapy based on the gene-expression similarity between LGG patients and melanoma patients treated with anti-PD1 and anti-CTLA4 therapy [28, 29].

### Construction of a LLPS-related signature

To identify the hub genes related to LLPS subtypes, WGCNA was performed on the expression profiles of prognostic LLPS-related DEGs by using the R package “WGCNA” [30, 31]. The optimal soft-thresholding power was selected according to the standard scale-free model fitting index R2. Then, we calculated the module eigengenes to investigate the correlations between the modules and LLPS subtypes. The hub genes in the modules most closely correlated to LLPS subtypes were identified. Interactions among these hub genes were visualized by using the STRING database (https://www.string-db.org/). To construct a LLPS-related prognostic signature, the hub genes were included into the LASSO Cox regression. Finally, the LPRS was calculated as follows:

$$ {\text{LPRS}} = \mathop \sum \limits_{i = 1}^{n} Coef_{i} *x_{i} $$

where $$x_{i} $$ and $$Coef_{i} $$ refer to the expression level of selected hub genes and corresponding LASSO coefficient, respectively. The prognostic value of the LPRS was evaluated by K–M survival curves with log-rank tests in all cohorts. The receiver operating characteristic (ROC) curve analyses were applied to assess the accuracy of LPRS in predicting OS of LGG patients. The independent prognostic value of LPRS was determined by univariate and multivariate Cox regression analyses. Moreover, we utilized the random-effects meta-analysis model to calculate the pooled hazard ratio (HR) of LPRS.

### The role of LPRS in two independent ICI therapy cohorts

To verify the role of LPRS in predicting the response to ICI therapy, two independent ICI therapy cohorts with available genomic and clinical information were included in our study: advanced urothelial cancer treated with atezolizumab, an anti-PD-L1 antibody (IMvigor210 cohort) [32], and metastatic melanoma with treatment of pembrolizumab, an anti-PD-1 antibody (GSE78220 cohort) [33]. We transformed the gene expression profiles into the TPM format for a higher comparability. The LPRS of each patient was calculated to evaluate its association with the response to ICI therapy.

### Cell lines and tissue samples

The normal human astrocyte cell line HA1800 and human glioma tumor cell lines U87, A172, LN229, U251 and U373 were purchased from Cell Bank of the Chinese Academy of Sciences. All cells were cultured in Dulbecco’s modified Eagle’s medium (DMEM) (Corning, USA) supplemented with 10% fetal bovine serum (FBS) (Gibco, USA) and 1% penicillin/streptomycin (P/S) in a humidified incubator with 5% carbon dioxide (CO_2_) at 37 °C. A total of fifteen clinical samples from low‐grade glioma patients were collected from May 2020 to October 2021 at the Neurosurgery Department of Wuhan Union Hospital, including 7 samples of grade II and 8 samples of grade III. During the same period, ten acute brain injury patient samples were selected as the control group in this study. Clinical information for these samples is outlined in Additional file 11: Table S1. This study was approved by the Ethics Committee of the hospital, and written informed consent was obtained from each patient.

### Quantitative real-time polymerase chain reaction (qRT-PCR) and Immunohistochemistry (IHC)

Briefly, total RNA was extracted from tissues and cell lines using RNAiso Plus (Takara 9109). According to the manufacturer instruction, cDNA was synthesized by reverse transcription using HiScript® III RT SuperMix for qPCR (+gDNA wiper) (Vazyme R323-01). The qRT-PCR assays were detected using the AceQ® qPCR SYBR Green Master Mix (Vazyme Q111-02) with PCR LightCycler480 (Roche Diagnostics, Basel, Switzerland). All expression data was normalized to β-actin as an internal control using the 2^–ΔΔ*Ct*^ method. All primers used were chemically synthesized by GeneCreate Biological Engineering Co. Ltd. (Wuhan, China). Then, we validated the protein level of selected LLPS-related hub genes by IHC experiment. Paraffin‐embedded clinical tissue specimens were sectioned, dewaxed, dehydrated, and washed with 3% methanol H_2_O_2_. The 3% bovine serum albumin (BSA) was incubated in phosphate buffer saline (PBS) for 30 min to block non-specific binding. Subsequently, the sections were incubated overnight at 4 °C using primary antibodies against FAM204A, SMUI, TNPO1 and TOP2A. Then, these sections were treated with three 5-min mild washing in PBS, followed by staining with secondary antibody (HRP polymer) for 50 min. After diaminobenzidine addition, the sections were then counterstained using hematoxylin, and blued in 1% ammonia water. Finally, the samples were sealed, viewed and photographed by light microscope. The intensity of positive staining of FAM204A, SMU1, TNPO1 and TOP2A in glioma and non-tumor brain tissue sections were measured through Image-Proplus 6.0 software. All the images were taken using the same microscope and camera sets. The intensity of positive staining in tissue sections was analyzed by average optic density per stained area (μm^2^) (IOD/area) for positive staining. The specific information of tissue samples used for qRT-PCR and IHC is listed in Additional file 11: Table S1**.** Primers and Antibodies can be found in Additional file 12: Table S2 and Additional file 13: Table S3 respectively.

### Statistical analysis

PERL programming language (version 5.32.0) was used to preprocess the RNA-seq data. R software (version 4.0.2) were applied for all statistical analyses and graph visualizations. The details about the versions and arguments/parameters of important 'R' packages in this work were listed in Additional file 14: Table S4. The Chi-square test was executed for the comparison of categorical variables between groups. Statistical significance for continuous variables between two groups or more than two groups was estimated by Student t test or the Kruskal–Wallis test, respectively. The correlation between two parameters was assessed through the Spearman correlation analysis. Two-tailed *P* < 0.05 was considered statistically significant.

## Results

### LLPS-related genes

**Fig. 2:**
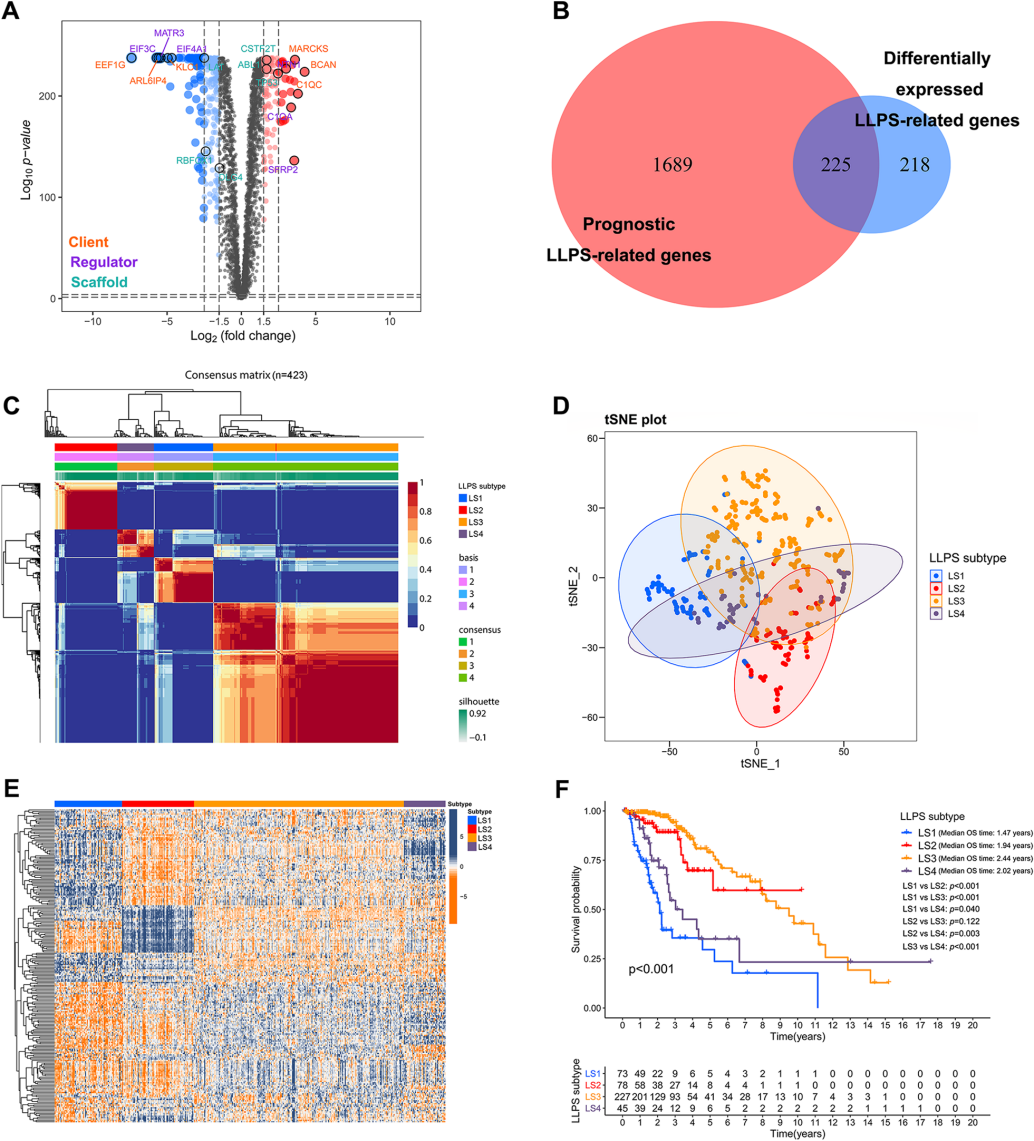
Identification of LLPS subtypes of LGG by using NMF algorithm. **A** Volcano plot showed DEGs (*P* < 0.05 and |log_2_FC|> 1.5) between LGG tissues in TCGA cohort and normal brain tissues in GTEx database. The gene names with top three log_2_FC and lower three log_2_FC were highlighted in the groups of clients, regulators and scaffolds respectively. **B** Venn diagram identified 225 prognostic LLPS-related DEGs. **C** Consensus map of NMF clustering. **D** tSNE plot for the expression profiles of 225 prognostic LLPS-related DEGs to distinguish LLPS subtypes. **E** Heatmap showed the expression levels of 225 prognostic LLPS-related DEGs among LLPS subtypes. **F** Kaplan–Meier survival analysis exhibited significantly different OS among LLPS subtypes

The detailed information of 3585 LLPS-related genes in homo sapiens was obtained from the DrLLPS, of which 85 were scaffolds (2.37%), 355 were regulators (9.9%) and 3145 were clients (87.73%; Additional file 1: Fig. S1A and Additional file 15: Table S5). Then, the transcriptome data of these 3585 LLPS-related genes were obtained from TCGA cohort and the GTEx database. The heatmap showed an obvious distinction in the expression of LLPS-related genes between LGG samples and normal samples (Additional file 1: Fig. S1B). Further differential expression analysis identified 443 LLPS-related DEGs, of which 170 were upregulated and 273 were downregulated in LGG samples compared with normal samples (Fig. 2A and Additional file 16: Table S6). By intersecting these DEGs with the prognostic LLPS-related genes obtained through univariate Cox regression analysis (Additional file 17: Table S7), 225 prognostic LLPS-related DEGs were identified (Fig. 2B), of which 3 were scaffolds, 24 were regulators and 198 were clients (Additional file 1: Fig. S1C). The top 10 significantly enriched GO terms and KEGG pathways for these prognostic LLPS-related DEGs were shown in Fig. S1D and E (see Additional file 1).

### Identification of LLPS subtypes in TCGA cohort

Based on the expression profiles of 225 prognostic LLPS-related DEGs, we performed NMF to identify LLPS subtypes in TCGA cohort. We selected 4 as the optimal clustering number, which was decided by the cophenetic, dispersion and silhouette indicators (Additional file 2: Fig. S2). Then, a total of 423 LGG patients were categorized into four subtypes (Fig. 2C), namely LS1 (n = 73), LS2 (n = 78), LS3 (n = 227) and LS4 (n = 45). The tSNE showed robust differences in distribution among four LLPS subtypes (Fig. 2D). The prominent differences in the expression of 225 prognostic LLPS-related DEGs were also observed in the heatmap (Fig. 2E). The K-M survival curve revealed that there was distinct survival difference among four LLPS subtypes (Fig. 2F). LS1 had the worst survival outcome, whereas LS3 had the best survival outcome.

**Fig. 3:**
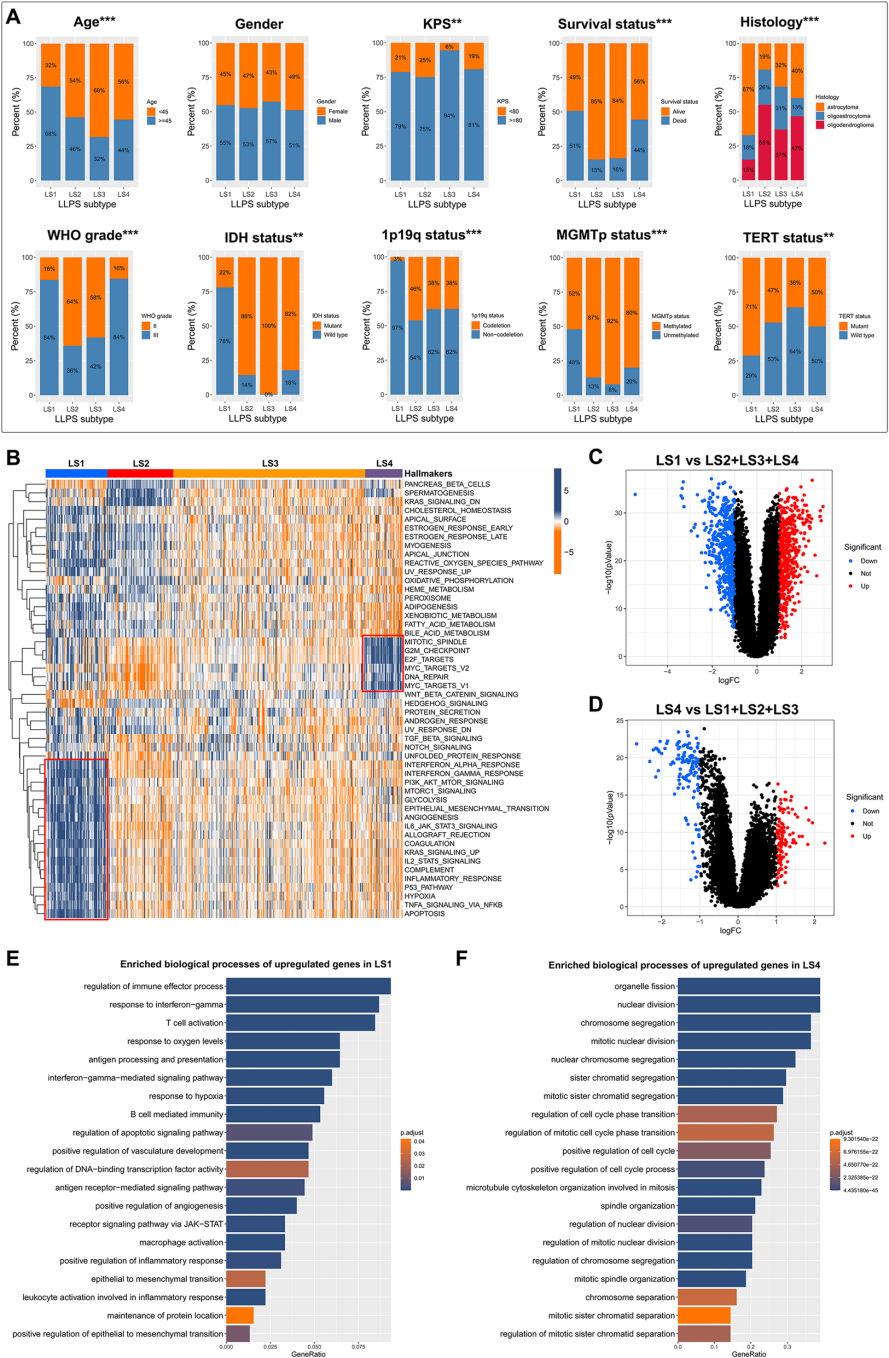
The comparisons of demographics, clinicopathological features and cancer hallmarks among LLPS subtypes. **A** Comparisons of age, gender, KPS, survival status, histology, WHO grade, IDH status, MGMTp status and TERT status among LLPS subtypes. **B** Heatmap illustrated the ssGSEA Z-scores of 50 hallmarks among LLPS subtypes. Blue represented high scores, and yellow represented low scores. The obvious differences were highlighted by red box. **C-D** Volcano plots showed DEGs (*P* < 0.05 and |log_2_FC|> 1.5) in LS1 and LS4 subgroups. **E–F** Gene Ontology enrichment analysis for significantly upregulated genes in LS1 and LS4, respectively. **P < 0.01, and ***P < 0.001

Subsequently, we compared the demographics and clinicopathological features of LGG patients in four LLPS subtypes. As illustrated in Fig. 3A, LS1 had more patients with age greater than or equal to 45 years compared with other subtypes. There were no significant differences among subtypes regarding gender distribution. The proportion of patients with Karnofsky Performance Score (KPS) greater than or equal to 80 was higher in LS3 than other subtypes. A higher percentage of deaths was observed in LS1 and LS4. Astrocytoma was more common in LS1, but oligodendroglioma was more common in other three subtypes. LS1 and LS4 had a significantly higher proportion of World Health Organization (WHO) grade III glioma compared with LS2 and LS3. There were also significant differences in molecular pathology among four LLPS subtypes. LGG patients with IDH wild type, or 1p19q non-codeletion, or MGMT promoter (MGMTp) unmethylated, or TERT mutant were more frequent in LS1.

To explore the underlying molecular mechanism related to the LLPS subtypes of LGG, we performed ssGSEA based on the transcriptome data of 50 gene sets retrieved from MSigDB. The ssGSEA Z-score was applied to quantify the levels of 50 hallmarks, and was visually illustrated by the heatmap (Fig. 3B). Compared with LS2 and LS3, LS1 and LS4 was more correlated with the hallmarks related to cell cycle and DNA repair, especially LS4, which may suggest an active proliferation of cancer cells. In addition, LS1 was positively associated with many cancer-related hallmarks, including glycolysis, epithelial mesenchymal transition (EMT), angiogenesis, hypoxia, apoptosis, inflammation and immunity. For further validation, we screened out a total of 507 upregulated genes (log_2_FC > 1 and *P* < 0.05) in LS1, and 121 upregulated genes (log_2_FC > 1 and *P* < 0.05) in LS4 (Fig. 3C, D), which were subsequently submitted to GO enrichment analyses. The enriched biological processes of upregulated genes in LS1 and LS4 were consistent with the results of ssGSEA (Fig. 3E, F). The above results might explain the poor survival of LS1 and LS4 to some extent.

### Comprehensive analyses of genomic alterations among LLPS subtypes

**Fig. 4:**
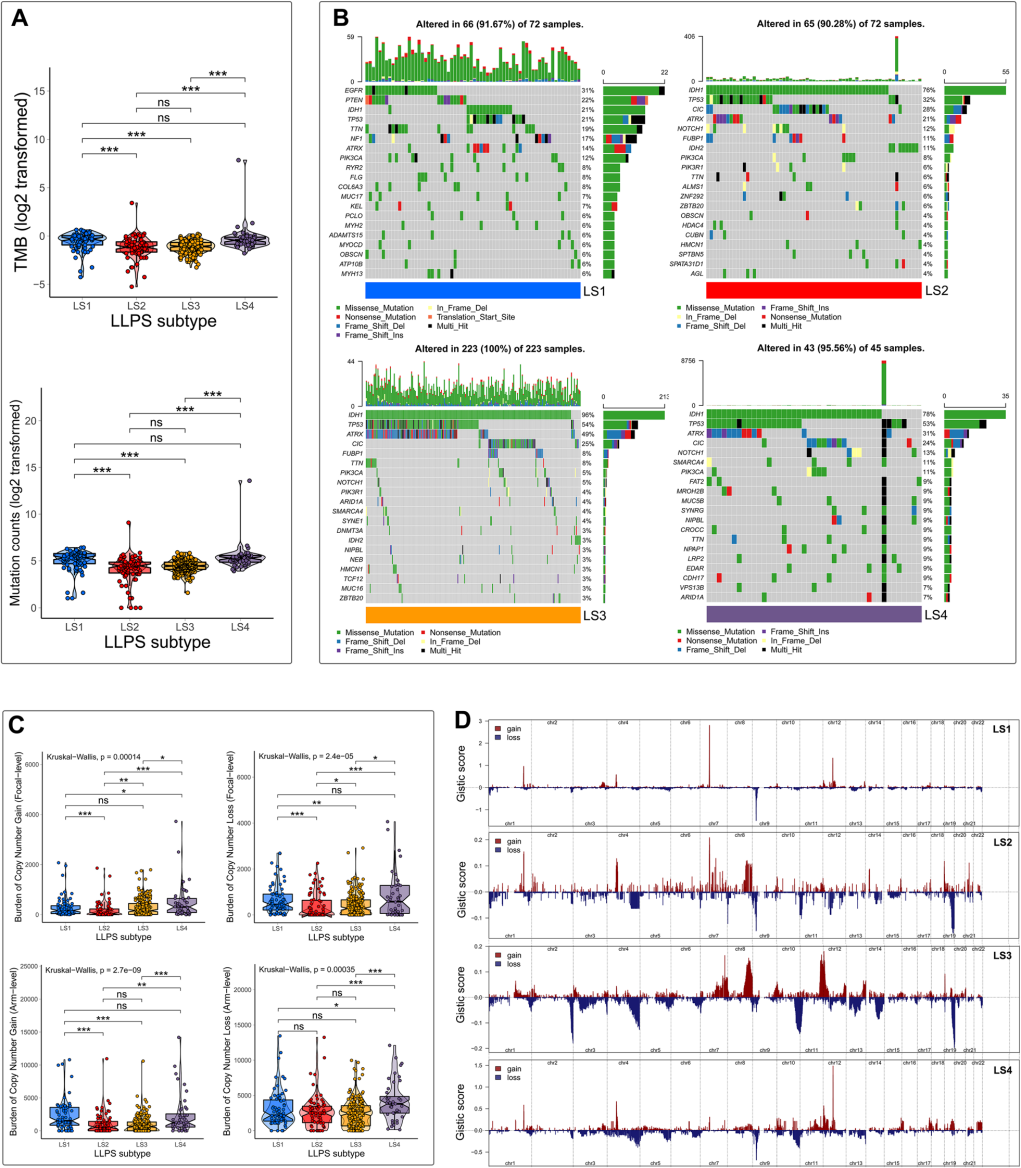
Comprehensive analyses of genomic alterations among LLPS subtypes. **A** Comparisons of TMB and mutation counts among LLPS subtypes. **B** Mutation profiles of LLPS subtypes. **C** Comparisons of CNA burdens at focal and arm levels among LLPS subtypes. **D** Copy number profiles for LLPS subtypes showed gains and losses of copy numbers of genes, which were placed based on their location on chromosomes, ranging from chromosome 1 to chromosome 22. * *P* < 0.05, ** *P* < 0.01, *** *P* < 0.001, and ^ns^ No significance

To gain a further insight into the disparity in the genomic layer, we compared the somatic mutation profiles and CNA landscapes among LLPS subtypes. First, LS1 and LS4 had significantly higher TMB and mutation counts than LS2 and LS3 (Fig. 4A). The somatic mutation profiles revealed that LS1 possessed specific top mutated genes compared with other three LLPS subtypes (Fig. 4B). EGFR was the most commonly mutated gene in LS1, followed by PTEN, whereas IDH1 and TP3 were the most two frequently mutated genes in LS2, LS3 and LS4. Next, we also noticed that there were clear differences in the degree of CNA burden among LLPS subtypes (Fig. 4C). It showed a trend that compared with LS2 and LS3, LS1 and LS4 had relatively higher burdens of gain and loss at both focal and arm levels. The distribution of Gistic score across all autosomes in LLPS subtypes was visualized in Fig. 4D. The results described above demonstrated an active genomic alteration in LS1 and LS4, which was likely due to their stronger proliferation ability of cancer cells.

### TIME and immunotherapeutic responses in different LLPS subtypes

**Fig. 5:**
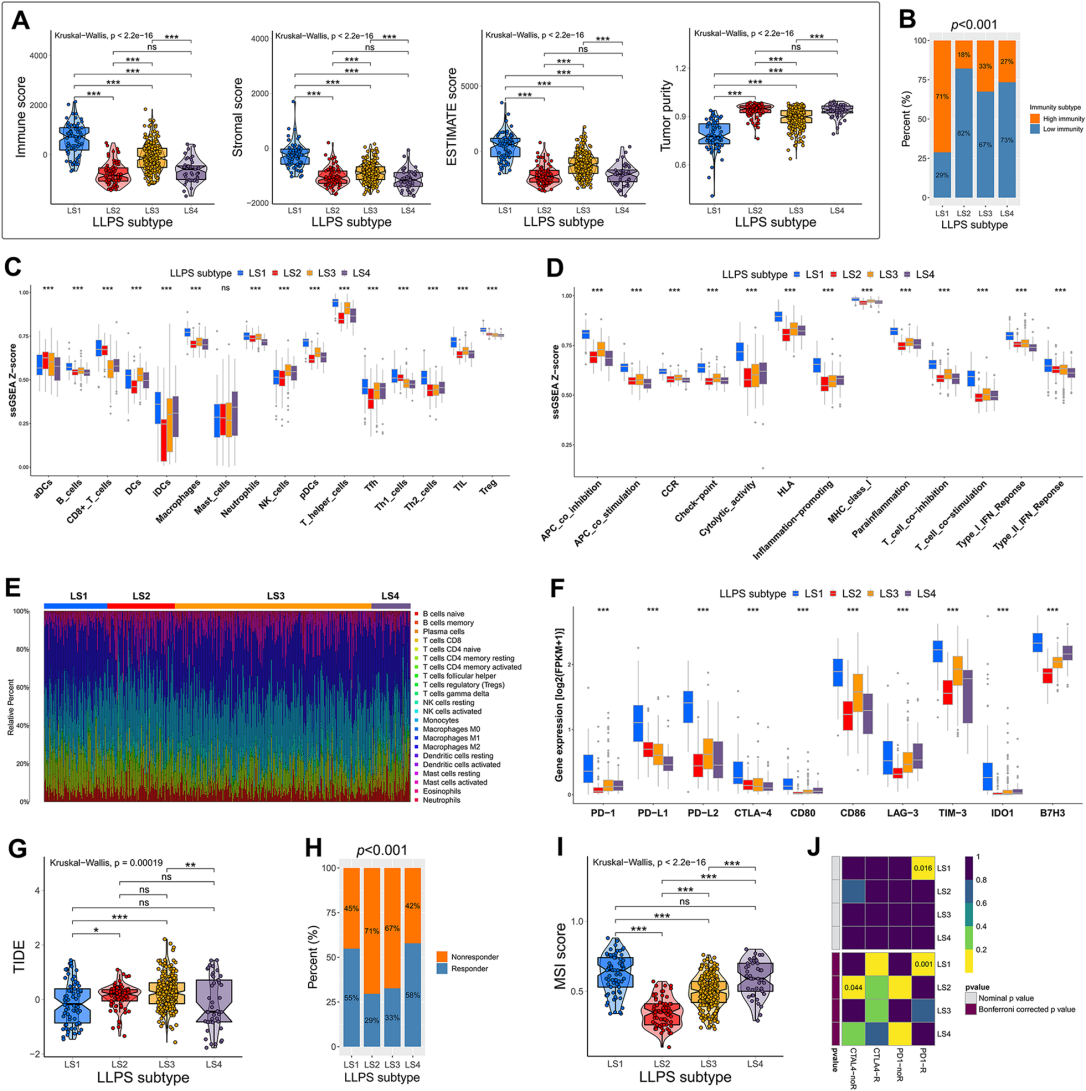
Different TIME patterns and immunotherapeutic responses of LLPS subtypes. **A** Comparison of immune scores, stromal scores, ESTIMATE scores and tumor purity among LLPS subtypes. **B** Different proportion of high and low immunity tumors among LLPS subtypes. **C**, **D** The levels of immune cell infiltrations and immune functions quantified by ssGSEA Z-score among LLPS subtypes. **E** The proportion of 22 immune cells quantified by CIBERSORT algorithm among LLPS subtypes. **F** Comparison of immune checkpoint expressions among LLPS subtypes. **G** Comparison of TIDE scores among LLPS subtypes. **H** The proportion of ICI therapy responders predicted by TIDE algorithm among LLPS subtypes. **I** Comparison of MSI scores among LLPS subtypes. **J** Subclass mapping analysis for predicting the likelihood of response to ICI therapy of LLPS subtypes. * *P* < 0.05, ** *P* < 0.01, *** *P* < 0.001, and ^ns^ No significance

Growing studies have begun to characterize the potential role of LLPS in regulating TIME and sensitivity to immunotherapy [19, 34]. Hence, we tried to compare the TIME patterns and immunotherapeutic responses among different LLPS subtypes. The ESTIMATE algorithm was firstly performed to quantify the constituents of the TIME of LGG. The results revealed that LS1 had highest immune, stromal, and ESTIMATE scores, and lowest tumor purity compared with other three subtypes. An opposite trend was observed in LS2 and LS4 (Fig. 5A). Then, LGG patients were classified into high-/low- immunity subtypes based on the ssGSEA Z-scores of 29 immune signatures. LS1 consisted of more proportions of high-immunity subtype, whereas LS2, LS3 and LS4 contained mainly low-immunity subtype (*P* < 0.001; Fig. 5B). The distribution of ssGSEA Z-score of 29 immune signatures was presented in Fig. 5C and D. There were significant differences in immune cell infiltrations and immune functions among four LLPS subtypes. Most notably, LS1 showed higher infiltration of most immune cells, and more robust immune functions than other three subtypes. Another algorithm, CIBERSORT, was also utilized to described the proportion of 22 immune cells in different LLPS subtypes, which was displayed in Fig. 5E.

We then compared the expression levels of immune checkpoints among LLPS subtypes. Compared with other subtypes, LS1 had significantly higher expression levels of all ten immune checkpoints, including PD-1 and its ligands (PD-L1 and PD-L2), CTLA-4 and its ligands (CD80 and CD86), LAG-3, TIM-3, IDO1 and B7H3 (Fig. 5F). Currently, ICI therapy has undoubtedly been a very promising strategy of immunotherapy, which caused a major breakthrough in antitumor treatment. Thus, we further used the TIDE algorithm to predict the response to ICI therapy of different LLPS subtypes. Our results revealed that the TIDE score was significantly decreased in LS1 and LS4 (Fig. 5G). The proportions of responders in LS1 and LS4 were nearly twofold that in LS2 and LS3 subtypes (*P* < 0.001; Fig. 5H). We also observed that compared with LS2 and LS3, LS1 and LS4 had higher MSI scores, which has been considered to be an indicator of effective immunotherapy (Fig. 5I). Moreover, we performed subclass mapping analysis to predict the response to ICI therapy, including PD-1 and CTLA-4 inhibitors, of the four LLPS subtypes. LS1 was found to be more sensitive to PD-1 inhibitor (Bonferroni corrected *P* = 0.001), while LS2 showed no response to CTLA-4 inhibitor (Bonferroni corrected *P* = 0.044; Fig. 5J). All these findings suggested that the LLPS patterns of LGG might play a crucial role in regulating the TIME patterns and immunotherapeutic responses.

### Construction of a prognostic signature based on LLPS-related hub genes

**Fig. 6:**
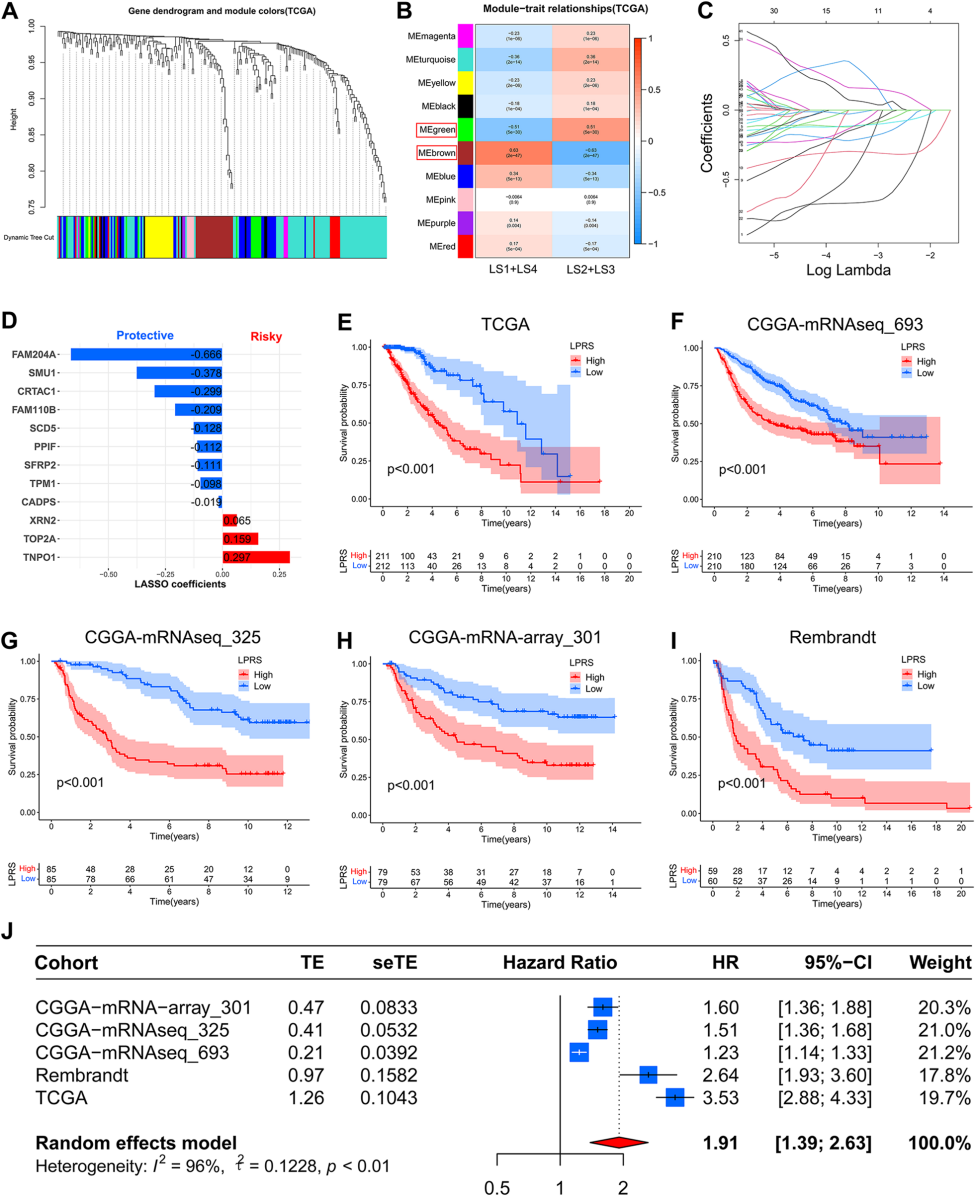
Construction of a LLPS-related prognostic signature for LGG patients. **A** Gene dendrogram and module colors. **B** Correlations of 10 modules with LLPS subtypes. The green model and brown model were selected and highlighted with red box. **C** LASSO regression analysis with minimal lambda value. **D** LASSO coefficients of selected LLPS-related genes. **E**–**I** The Kaplan–Meier survival curves of LPRS in TCGA, CGGA-mRNAseq_693, CGGA-mRNAseq_325, CGGA-mRNA-arry_301 and Rembrandt cohorts. **J** Meta-analysis with random-effects showed a pooled hazard ratio (HR) of LPRS

To identify the LLPS-related hub genes, WGCNA analysis was performed with the transcriptome data of 225 prognostic LLPS-related DEGs. We selected 2 as the optimal soft-thresholding power based on the standard scale-free model fitting index R2 (Additional file 3: Fig. S3A). Then, a total of 10 gene modules were obtained. Based on the previous findings, there were similarities in terms of survival, genomic alteration and immune characteristic between LS1 and LS4, also between LS2 and LS3. Thus, LS1 and LS4, and LS2 and LS3, were merged together, respectively. Among these 10 models, the brown module containing 26 genes exhibited the highest correlation with LS1 and LS4, and the green module containing 17 genes showed the highest correlation with LS2 and LS3 (Fig. 6A, B). Then, the genes in these two models were deemed as LLPS-related hub genes, and were picked for subsequent analyses. Fig. S3B and C (see Additional file 3) showed the top 10 enriched GO terms and KEGG pathways for the genes of the green and brown modules. As shown in the interaction networks, there were 15 genes and 11 edges in the green module, and 22 genes and 92 edges in the brown module with a threshold weight > 0.15 (Additional file 3: Fig. S3D, E).

Next, these forty-three LLPS-related hub genes were incorporated into the LASSO Cox regression in the TCGA cohort, twelve of which stood out for the construction of a LLPS-related prognostic signature (Additional file 3: Fig. S3F and Fig. 6C). Figure 6D exhibited the LASSO coefficients of each selected gene in this signature. There were nine protective genes and three risky genes for survival outcomes. The K-M survival curves of these 12 selected genes were shown in Fig. S4 (see Additional file 4). Then, the LPRS of each LGG patient was calculated by summing the product of the expression levels of each selected LLPS-related hub gene and corresponding LASSO coefficients. The median LPRS was used to stratify patients into high-LPRS and low-LPRS subgroups. As shown in the K-M survival curves, high-LPRS patients exhibited worse OS in TCGA cohort (Fig. 6E). Consistent results were obtained in other four independent cohorts (Fig. 6F–I). Additionally, the high accuracy of LPRS in predicting 1-, 3- and 5-year OS was confirmed by the ROC curves (Additional file 5: Fig. S5A). According to the univariate and multivariate Cox regression analyses, LPRS was an independent prognostic indicator for OS in all cohorts (Additional file 5: Fig. S5B). Further, meta-analysis was performed, and revealed that the overall pooled HR of LPRS was 1.91 (95% CI = 1.39–2.63; Fig. 6J).

### Correlation of LPRS with clinicopathological features, genomic alterations and TIME patterns

**Fig. 7:**
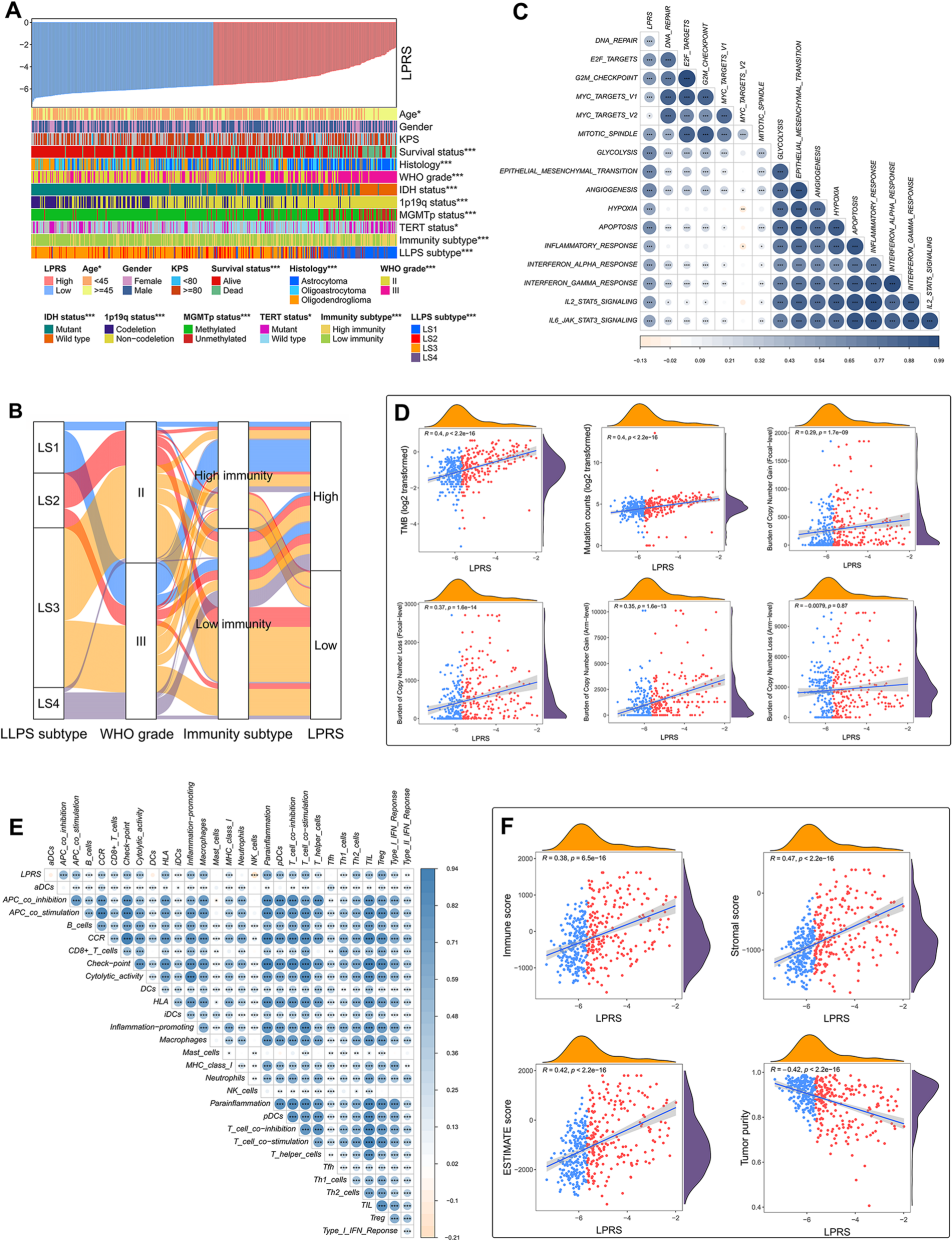
Correlation of LPRS with clinicopathological features, genomic alterations and TIME patterns in TCGA cohort. **A** An overview of the correspondence between LPRS and other features of LGG patients. **B** Alluvial diagram showed the attribute changes of LLPS subtypes, WHO grade, immunity subtypes and LPRS. **C** Correlation between LPRS and the known cancer hallmarks. **D** Correlation of LPRS with TMB, mutation counts, and copy number burdens at focal and arm levels. **E** Correlation between LPRS and the ssGSEA Z-scores of 29 immune signatures. **F** Correlation of LPRS with immune scores, stromal scores, ESTIMATE scores and tumor purity. * *P* < 0.05, ** *P* < 0.01, and *** *P* < 0.001

The prognostic value of LPRS has been well elucidated. Then, we sought to explore its clinical relevance in TCGA cohort. As shown in Fig. 7A, LPRS was ranked from low to high to show the correlation between LPRS and clinicopathological features. There were significant differences between high- and low-LPRS subgroups in terms of age, survival status, histology, WHO grade, IDH status, 1p19q status, MGMTp status, TERT status, immunity subtypes and LLPS subtypes. The attribute changes of each patient were visualized by an alluvial diagram (Fig. 7B). We also compared the levels of LPRS between subgroups stratified by different clinicopathological features. Patients with the clinicopathological features of age ≥ 45 years, death, astrocytoma, WHO grade III, IDH wild type, 1p19q non-codeletion and MGMTp unmethylated presented significantly higher levels of LPRS, whereas no LPRS differences were observed between patients stratified by gender, KPS and TERT status (Additional file 6: Fig. S6A). In addition, we found that the high-immunity subtype was associated with a higher LPRS than low-immunity subtype, and LPRS was ranked in increasing order for LS3, LS2, LS4 and LS1 (Additional file 6: Fig. S6). In other four independent cohorts, the correlation between LPRS and clinicopathological features was also analyzed and presented as heatmaps (Additional file 7: Fig. S7A–D).

To better characterize the LLPS-related prognostic signature, we tested the correlation between the cancer hallmarks and LPRS. A correlation heatmap revealed that LPRS was significantly positively correlated with many well-known hallmarks of cancer, including DNA repair and cell cycle-related hallmarks (Fig. 7C). As expected, further analyses demonstrated that LPRS was markedly positively linked with TMB, mutation counts, burden of copy number gain and loss at focal-level, and burden of copy number gain at arm-level (Fig. 7D). These data indicated to some extent that a higher LPRS represents a higher frequency of genomic alterations.

Given that LPRS was associated with different immunity subtypes, we took further insight into the detailed differences in TIME patterns as the LPRS changes. In TCGA cohort, the correlation between LPRS and 29 immune signatures was illustrated by a correlation heatmap (Fig. 7E). Then, LPRS was significantly positively correlated with the immune, stromal, and ESTIMATE scores, but negatively correlated with tumor purity, which indicating that the infiltration levels of immune and stromal cells increase with the elevation of LPRS (Fig. 7F). Further correlation analyses were carried out between LPRS and the infiltration levels of 22 immune cells quantified by the CIBERSORT algorithm. It turned out that LPRS was positively correlated with memory resting CD4 + T cells, M1 macrophages and Tregs, and was negatively correlated with activated mast cells and monocytes (Additional file 10: Fig. S10A). In addition, LPRS was observed to be positively correlated with the expression levels of ten immune checkpoints (Additional file 10: Fig. S10A). The above explorations regarding the correlation between LPRS and TIME patterns were also performed in other four independent cohorts (Additional file 8: Fig. S8, Additional File 9: Fig. S9, additional file 10: Fig. S10B–E). The results obtained were generally agreement with those of TCGA cohort.

### The role of LPRS in predicting the response to ICI therapy

**Fig. 8:**
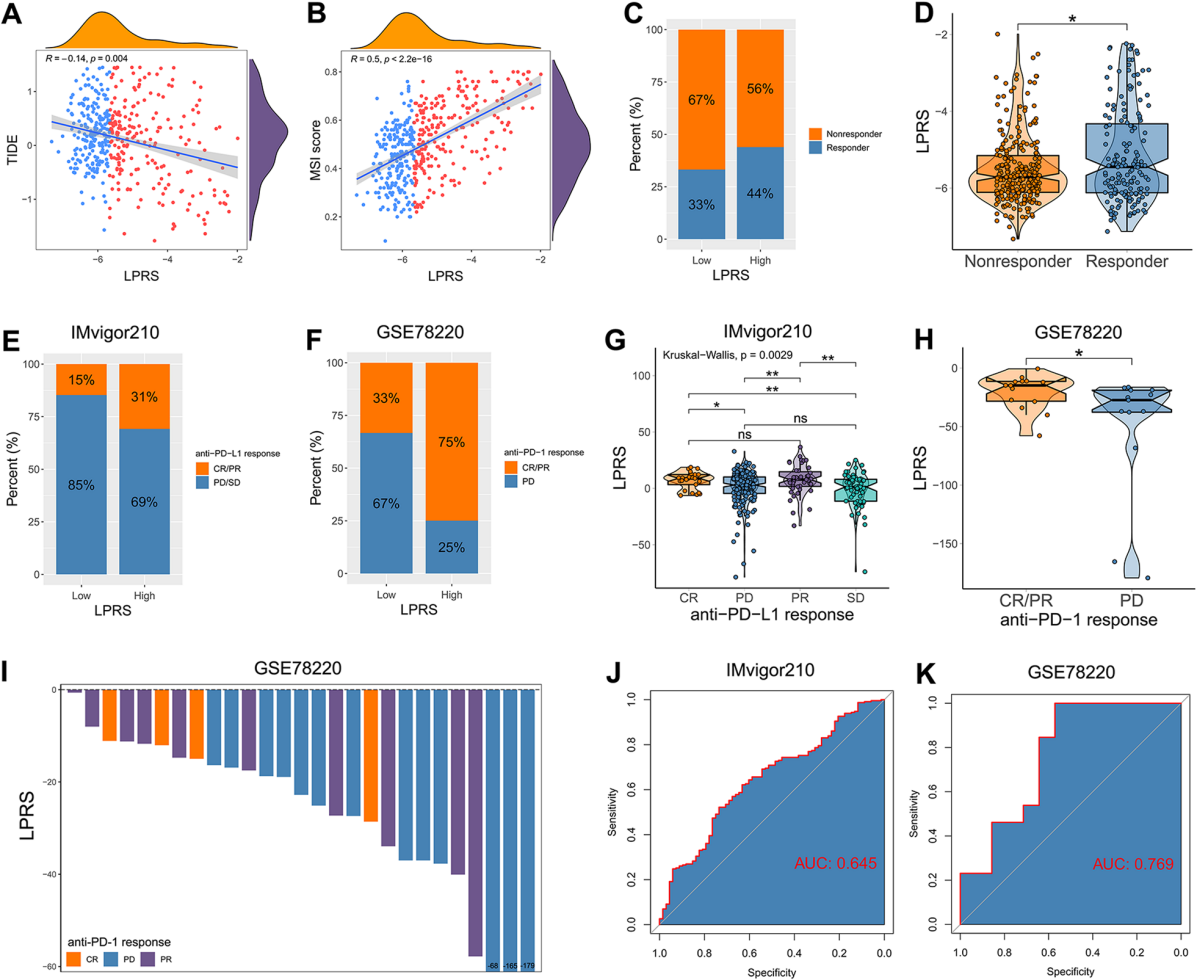
The role of LPRS in predicting the response to ICI therapy. **A, B** LPRS was correlated with TIDE score and MSI score in TCGA cohort. **C** The proportion of ICI therapy responders predicted by TIDE algorithm between high-LPRS and low-LPRS subgroups in TCGA cohort. **D** Comparison of LPRS levels between responders and non-responders in TCGA cohort. **E** The proportion of patients with response to anti-PD-L1 immunotherapy in IMvigor210 cohort (CR/PR vs. PD/SD: 31% vs. 69% in high-LPRS subgroup, CR/PR vs. PD/SD: 15% vs. 85% in low-LPRS subgroup; *P* = 0.001). **F** The proportion of patients with response to anti-PD-1 immunotherapy in GSE78220 cohort (CR/PR vs. PD/SD: 75% vs. 25% in high-LPRS subgroup, CR/PR vs. PD/SD: 33% vs. 67% in low-LPRS subgroup; *P* = 0.031). **G** Comparison of LPRS levels among the subgroups of different response to anti-PD-L1 immunotherapy in IMvigor210 cohort. **H**, **I** Comparison of LPRS levels among the subgroups of different response to anti-PD-1 immunotherapy in GSE78220 cohort. **J**, **K** The predictive power of LPRS in patients with anti-PD-L1/ anti-PD-1 immunotherapy (IMvigor210 cohort: AUC = 0.645; GSE78220 cohort: AUC = 0.769). *CR* complete response, *PR* partial response, *PD* progressive disease, *SD* stable disease. * *P* < 0.05, ** *P* < 0.01, and ^ns^ No significance

ICI therapy represented by PD-1, PD-L1 and CTLA-4 inhibitors has undoubtedly achieved encouraging progress in the therapeutic landscape of cancer. However, the considerable heterogeneity in therapeutic response has long been a major challenge to improve survival outcomes for glioma patients. Hence, we focused on the role of LPRS in predicting the response to ICI therapy. In TCGA cohort, patients with higher LPRS showed lower level of TIDE and higher level of MSI score (Fig. 8A, B). Based on TIDE algorithm, the high-LPRS subgroup contained a higher proportion of responders to ICI therapy compared with low-LPRS subgroup (Fig. 8C). Besides, the LPRS of responders was significantly higher than that of non-responders (Fig. 8D). Above all, we speculated that high-LPRS patients could benefit more from ICI therapy than low-LPRS patients. To make our findings more convincing, we next investigated whether the LPRS could predict patients’ response to ICI therapy in two independent ICI therapy cohorts, namely IMvigor210 (anti-PD-L1 cohort) and GSE78220 (anti-PD-1 cohort). In both cohorts, there were significantly higher proportion of complete response (CR) or partial response (PR) in the high-LPRS subgroup (Fig. 8E, F). Patients with the outcome of CR or PR exhibited significantly higher level of LPRS than patients with the outcome of stable disease (SD) or progressive disease (PD) (Fig. 8G, H). An overall satisfactory accuracy of LPRS for predicting the response to ICI therapy was confirmed by the ROC curves (Fig. 8J, K). Altogether, our findings strongly indicated that the LPRS was associated with the response to ICI therapy, and has the potential to serve as a response indicator in clinical practice.

### The expression levels of selected LLPS-related hub genes

**Fig. 9:**
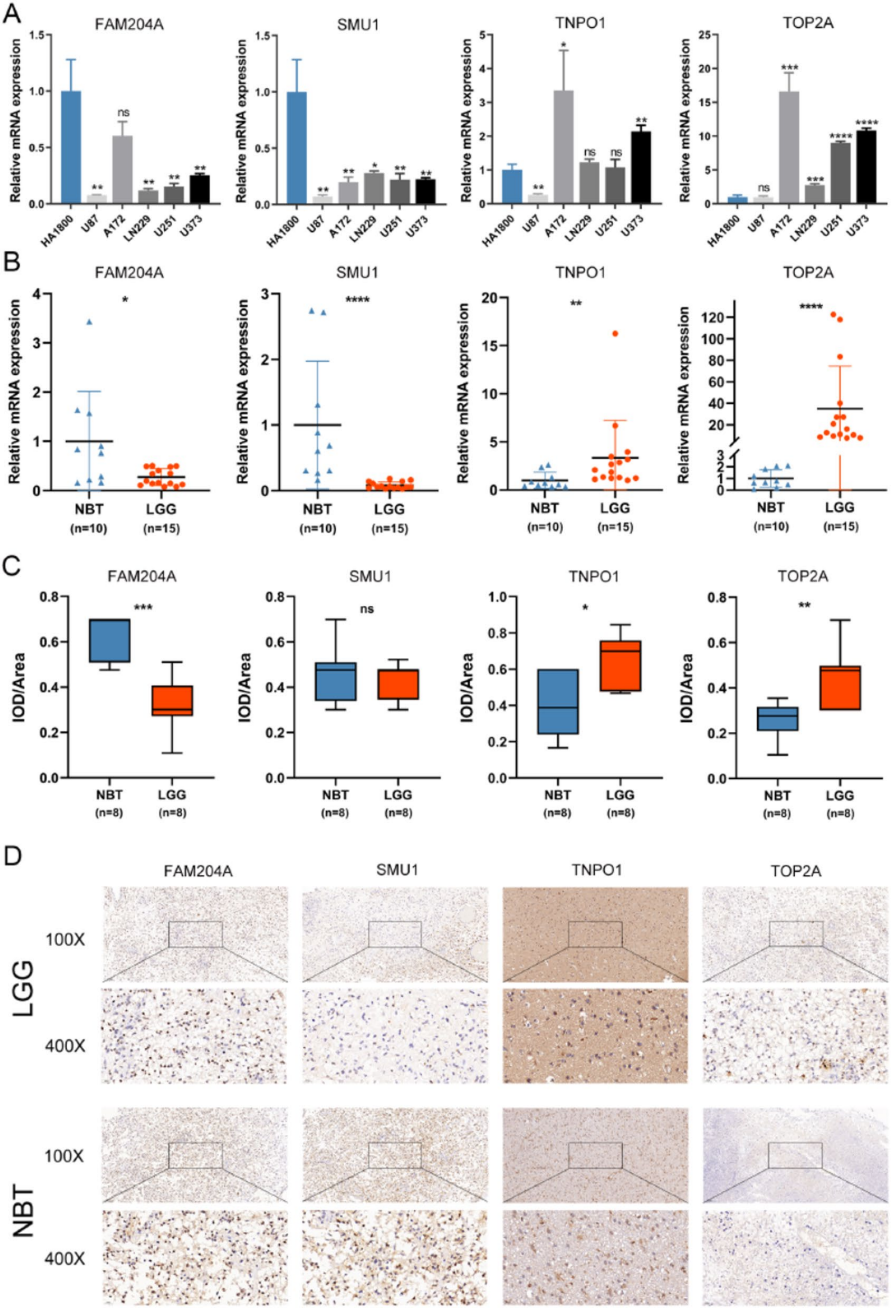
The expression levels of selected LLPS-related hub genes. **A** Scatter plots of differential transcript levels between FAM204A, SMU1, TNPO1 and TOP2A in glioma cell lines and normal human astrocytes cell lines (HA1800). **B** Scatter plots of differential transcript levels between FAM204A, SMU1, TNPO1 and TOP2A in LGG and NBT. **C** Comparison of the average IOD/Area of FAM204A, SMU1, TNPO1 and TOP2A between LGG and NBT. **D** Representative IHC staining images. *LGG* low-grade glioma, *NBT* non-tumor tissues, *IOD/area* Integrated optical density per stained area. *P < 0.05, **P < 0.01, ***P < 0.001, and ^ns^ No significance

We selected four LLPS-related hub genes (FAM204A, SMU1, TNPO1 and TOP2A) involved in LPRS to test their transcript levels in cell lines, LGG tissues and non-tumor brain tissues. The qRT-PCR results showed that the mRNA expression levels of FAM204A and SMU1 in human glioma cell lines overall showed a downward trend compared with the HA1800, while the mRNA expression levels of TNPO1 and TOP2A showed an overall upward trend (Fig. 9A). The qRT-PCR results of tissue samples are consistent with that of cell lines. Compared with non-tumor brain tissue, the transcription levels of FAM204A and SMU1 in LGG tissues decreased, while the transcription levels of NPO1 and TOP2A increased (Fig. 9B). Further, the protein expression level of these four LLPS-related hub genes was detected by IHC staining and analyzed by calculating the IOD/area. Compared with non-tumor tissues, FAM204A was down-regulated, but TNPO1 and TOP2A were up-regulated in glioma tissues. There was no significant difference in the protein level of SMU1 between LGG and NBT (Fig. 9C). Representative IHC staining images were shown in Fig. 9D.

## Discussion

Evidence is now mounting that LLPS process plays an integral role in the tumorigenesis and progression [17]. Moreover, the formation of different TIME patterns has also been shown to be correlated with LLPS due to its involvement in the regulation of immune signaling [34, 35]. Therefore, we supposed that a comprehensive exploration of LLPS-related biomarkers held great promise for the identification of novel subtypes of tumors, and the prediction of prognosis and immunotherapeutic response. In this study, we exclusively focused on LGG patients. Based on the expression profiles of 225 prognostic LLPS-related DEGs, we identified four LLPS subtypes of 423 LGG patients by using NMF algorithm. Then, significant differences among four LLPS subtypes were observed regarding prognosis, clinicopathological features, cancer hallmarks, genomic alterations, TIME patterns and immunotherapeutic responses. To make individualized integrative assessments, a prognostic signature, namely LPRS, was constructed via the WGCNA algorithm and LASSO Cox regression. Results revealed that LPRS was correlated with prognosis, clinicopathological features, genomic alterations and TIME patterns of LGG patients. The predictive power of LPRS in response to ICI therapy was also prominent.

Representative hallmarks of tumors include sustained proliferation, angiogenesis, EMT and genome rearrangements, and so on. How do tumors acquire these hallmark characteristics? In recent years, the field of LLPS is changing the way researchers and clinicians are now thinking about the acquisition of malignant characteristics of tumors [17]. For instance, MYC has the potential to form phase-separated transcription condensates by binding to super-enhancers, which can lead to the expression of VEGF and promote angiogenesis [36]. The LLPS of transcriptional coactivators, YAP and TAZ, is involved in the activation of EMT [37–39]. The abnormal LLPS of ENL is enriched in genomic loci of chromosomes, and recruits large numbers of related transcription complexes, resulting in genome rearrangements in cancer [40, 41]. In this study, different LLPS subtypes of LGG patients exhibited distinct tumor hallmarks characteristics quantized by ssGSEA. The LS1 subtype was characterized by glycolysis, angiogenesis, EMT, hypoxia-responsive activation and regulation of apoptotic signaling pathway. However, the critical tumor hallmarks of LS4 were related to cell cycle and genome stability, which corresponded with the active genomic alteration of LS4. Compared with LS2 and LS3, LS1 and LS4 showed significant malignant progression features, which provided a possible explanation for the worse prognosis of LS1 and LS4. Simultaneously, the constructed LPRS also showed a significant correlation with these well-known hallmarks of tumors. These results provided compelling support for the nonnegligible role of LLPS in conferring specific hallmarks of tumors.

The classical view held that tumors could be divided into three different TIME patterns: immune-inflamed, immune-excluded, and immune-desert. It has long been known that glioma is dominated by immune-excluded and immune-desert patterns, which contribute, to a large extent, to the immune escape of glioma cells and the immunotherapy resistance of patients. The formation of specific TIME pattern is an immensely complex process involving numerous factors. Recent reports about the role of LLPS in innate and adaptive immunity shed new light on this filed. For example, the LLPS of cyclic GMP-AMP synthase (cGAS) promotes the secondary messenger cyclic GMP-AMP (cGAMP) production and innate immune signaling [42]. A large proportion of biomolecules in the transmembrane signaling receptors of T cells might phase separate into clusters to facilitate the transduction of signals and regulate immune responses of tumors [43]. In this study, LS1 had higher immune scores and stroma scores, but lower tumor purity compared with other subtypes, indicating that LS1 was surrounded by more nontumor components. Furthermore, LS1 displayed the activation of adaptive immune pathway and the infiltration of tumor infiltrating lymphocyte infiltration. Thus, it can be considered that LS1 corresponded to the immune-inflamed pattern, and was likely to respond well to immunotherapy. Subsequent prediction of the response to ICI therapy confirmed this speculation. Based on the TIDE algorithm, LS1 presented relatively lower TIDE score and higher MSI score compared with LS2 and LS3. In addition, subclass mapping analysis revealed that LS1 responded remarkably well to PD-1 inhibitor. Taking together, these findings strongly suggested that the established LLPS subtypes would contribute to the differential recognition of TIME patterns, and help to identify patients suitable for ICI therapy. Follow-up studies are warranted to determine the detailed mechanism of how LLPS processes regulate the specific formation of TIME patterns.

Given the multifaceted heterogeneities among four LLPS subtypes, we considered that it was feasible to construct a prognostic signature for the quantification of such heterogeneities, and also for the individualized integrative assessments. As expected, the constructed LPRS not only exhibited a close correlation with clinicopathological features, representative cancer hallmarks, genomic alterations and TIME patterns of LGG patients, but also possessed a prominent power in predicting prognosis and response to ICI therapy. LPRS was composed of twelve selected LLPS-related genes, of which two were regulators and ten were clients. The regulator TNPO1, also known as Karyopherin-β2, has been reported to inhibit the LLPS of an RNA-binding protein Fused in Sarcoma (FUS) and escort it into the nucleus [44]. Another regulator, SFRP2, is required for P-body assembly [26]. Of these ten clients, CADPS, CRTAC1, SCD5 and TPM1 can formed postsynaptic density [45]. FAM204A and PPIF are involved in nucleolus [46–48]. SMU1 participates in the formation of stress granule [49]. Other three clients can form variety types of biomolecular condensates (FAM110B: centrosome, spindle pole body; TOP2A: nucleolus, centrosome, spindle pole body and P-body; XRN2: nucleolus, P-body and stress granule) [48, 50–58]. As is already evident, what we currently know about these LLPS-related genes is almost exclusively confined to the forming types of biomolecular condensates that they are involved in. Thus, a more in-depth mechanism of how the LLPS processes that underlie the assembly of various biomolecular condensates affect the occurrence and development of tumors needs to be investigated in the future.

Up to now, there have been so many classifications of LGG patients based on classical biomarkers or star molecules related to a specific topic. For example, the IDH1 mutation status has long been recognized as a very important prognostic biomarker for glioma. Although the LLPS subtypes showed different distribution of IDH1 mutation status, we didn’t think the survival differences among LLPS subtypes were due to such difference. It could be seen that LS2 and LS4 had similar IDH1 mutation ratio, but the prognosis of LS4 was significant worse than that of LS2. Compared with previous classifications of LGG patients, the advantage of our LLPS subtyping was showing multi-dimensional heterogeneities, including prognosis, clinicopathological features, cancer hallmarks, genomic alterations, TIME patterns and immunotherapeutic responses, especially immunotherapeutic responses, a topic which is of great clinical interest. Nonetheless, several limitations of this study should be addressed. First, all analyses were performed based on the retrospective data of public databases, using the prospective multi-center cohorts will produce more reliable results. Second, due to the limitation of immunotherapy cohorts with publicly available transcriptional data and clinical information, we could only assess the predictive power of LPRS in response to ICI therapy by using the cohorts of urothelial cancer and metastatic melanoma. Finally, bioinformatic analyses are not able to deeply elucidate the molecular mechanisms, experimental evidences are indispensable to further exploration.

## Conclusion

Taken together, we divided LGG patients into four LLPS subtypes with distinct prognosis, clinicopathological features, cancer hallmarks, genomic alterations, TIME patterns and immunotherapeutic responses. In addition, a prognostic signature, LPRS, was proposed for individualized integrative assessment. The findings might facilitate individualized prognosis prediction and better immunotherapy options for LGG patients, and further studies are needed to clarify this point.

## Supplementary Information

**Additional file 1: Fig. S1**. The composition and functional enrichment of LLPS-related genes in LGG patients. A The fractions of scaffolds, regulators and clients with available gene-expression data in TCGA cohorts. B The expression levels of 3585 LLPS-related genes in LGG tissues from TCGA cohort compared with normal brain tissues from GTEx database. C The distribution of scaffolds, regulators and clients among 225 prognostic LLPS-related DEGs. D-E The top 10 significantly enriched GO terms and KEGG pathways for 225 prognostic LLPS-related DEGs.

**Additional file 2: Fig. S2**. The association between cophenetic, dispersion, evar, residuals, rss, silhouette, and sparseness concerning the clustering numbers.

**Additional file 3: Fig. S3**. Identification of LLPS-related hub genes to construct a a prognostic signature. A The scale independence and mean connectivity plot for selecting soft threshold. B-C The top 10 significantly enriched GO terms and KEGG pathways for the genes in the green and brown modules. D-E The network of the genes in the green and brown modules (weight of edge > 0.15). F Cross-validation for tuning parameter selection in the proportional hazards model.

**Additional file 4: Fig. S4**. The Kaplan–Meier survival curves of 12 selected LLPS-related genes in TCGA cohort.

**Additional file 5: Fig. S5**. The prognostic value of LPRS in multiple cohorts. A ROC curve analyses of LPRS in predicting 1-, 3- and 5-year OS in TCGA, CGGA-mRNAseq_693, CGGA-mRNAseq_325, CGGA-mRNA-arry_301 and Rembrandt cohorts. B The independent prognostic value of LPRS was validated by performing univariate and multivariate Cox regression analyses in TCGA, CGGA-mRNAseq_693, CGGA-mRNAseq_325, CGGA-mRNA-arry_301 and Rembrandt cohorts.

**Additional file 6: Fig. S6**. The correlations of LPRS with clinicopathological features of LGG patients in TCGA cohort. **P < 0.01, ***P < 0.001, and ns No significance.

**Additional file 7: Fig. S7**. Heatmaps showing the correlations of LPRS with clinicopathological features of LGG patients in CGGA-mRNAseq_693 cohort, CGGA-mRNAseq_325 cohort, CGGA-mRNA-arry_301 cohort and Rembrandt cohort. *P < 0.05, **P < 0.01, and ***P < 0.001.

**Additional file 8: Fig. S8**. Correlations between LPRS and the ssGSEA Z-scores of 29 immune signatures in CGGA-mRNAseq_693 cohort, CGGA-mRNAseq_325 cohort, CGGA-mRNA-arry_301 cohort and Rembrandt cohort. *P < 0.05, **P < 0.01, and ***P < 0.001.

**Additional file 9: Fig. S9**. Correlations of LPRS with immune scores, stromal scores, ESTIMATE scores and tumor purity in CGGA-mRNAseq_693 cohort, CGGA-mRNAseq_325 cohort, CGGA-mRNA-arry_301 cohort and Rembrandt cohort.

**Additional file 10: Fig. S10**. Correlations of LPRS with the infiltration levels of 22 immune cells and the expression levels of immune checkpoints in TCGA cohort, CGGA-mRNAseq_693 cohort, CGGA-mRNAseq_325 cohort, CGGA-mRNA-arry_301 cohort and Rembrandt cohort.

**Additional file 11: Table S1**. Clinical features of tissues used for qRT-PCR and IHC.

**Additional file 12: Table S2**. The primers sequence used for qRT-PCR.

**Additional file 13: Table S3**. The primary antibodies used in immunohistochemistry (IHC).

**Additional file 14: Table S4**. The details about the versions and arguments/parameters of important 'R' packages in this work.

**Additional file 15: Table S5**. The detailed information of 3585 LLPS-related genes in homo sapiens.

**Additional file 16: Table S6**. The LLPS-related DEGs between LGG samples and normal samples.

**Additional file 17: Table S7**. The prognostic LLPS-related genes obtained through univariate Cox regression analysis in TCGA cohort.

## Data Availability

Publicly available datasets were analyzed in this study. This data can be found here: the data analyzed in this study can be acquired in the DrLLPS, (http://llps.biocuckoo.cn/), TCGA (https://portal.gdc.cancer.gov/), CGGA (http://www.cgga.org.cn/), Rembrandt (http://gliovis.bioinfo.cnio.es/) and GTEx (https://gtexportal.org/home/) websites.

## Abbreviations

IDRs: Intrinsically disordered domains
LLPS: Liquid–liquid phase separation
P-body: Processing body
LGG: Lower-grade glioma
TCGA: The Cancer Genome Atlas
TIME: Tumor immune microenvironment
LPRS: LLPS-related prognostic risk score
ICI: Immune checkpoint inhibitor
RNA-seq: RNA sequencing
CGGA: Chinese Glioma Genome Atlas
OS: Overall survival
GTEx: Genotype-Tissue Expression
FPKM: Fragments per kilobase of exon model per million mapped reads
DrLLPS: Data resource of LLPS
DEGs: Differentially expressed genes
GO: Gene Ontology
KEGG: Kyoto Encyclopedia of Genes and Genomes
NMF: Non-negative matrix factorization
tSNE: T-distributed stochastic neighbor embedding
K-M: Kaplan–Meier
MSigDB: Molecular Signatures Database
ssGSEA: Single sample gene set enrichment analysis
MAF: Mutation annotation format
TMB: Tumor mutation burden
CNA: Copy number alteration
TIDE: Tumor Immune Dysfunction and Exclusion
MSI: Microsatellite instability
WGCNA: Weighted gene co-expression network analysis
LASSO: Least absolute shrinkage and selection operator
ROC: Receiver operating characteristic
HR: Hazard ratio
DMEM: Dulbecco’s modified Eagle’s medium
FBS: Fetal bovine serum
P/S: Penicillin/streptomycin
CO_2_: Carbon dioxide
qRT-PCR: Quantitative real-time polymerase chain reaction
NBT: Non-tumor brain tissues
IOD: Integrated optical density
KPS: Karnofsky Performance Score
WHO: World Health Organization
MGMTp: MGMT promoter
EMT: Epithelial mesenchymal transition
CR: Complete response
PR: Partial response
SD: Stable disease
PD: Progressive disease
cGAS: Cyclic GMP-AMP synthase
cGAMP: Cyclic GMP-AMP
FUS: Fused in Sarcoma
