# Supplementary figures and images for: An integrative multi-omics analysis based on liquid–liquid phase separation delineates distinct subtypes of lower-grade glioma and identifies a prognostic signature
Source: J Transl Med. 2022 Jan 29;20:55. doi: 10.1186/s12967-022-03266-1 (PMC8800244; doi:10.1186/s12967-022-03266-1)

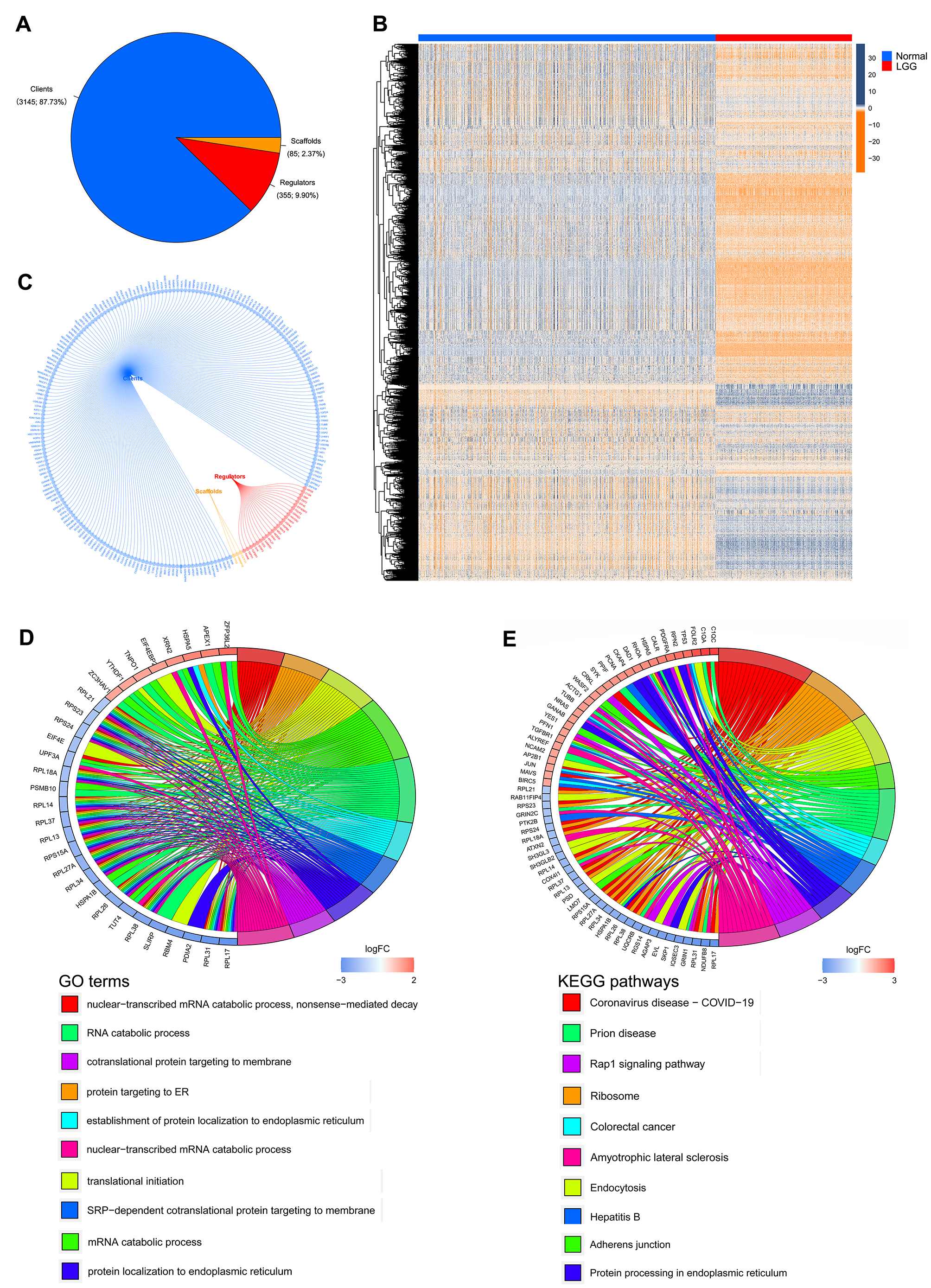

Supplement: Supplementary file 1 — Additional file 1: Fig. S1. The composition and functional enrichment of LLPS-related genes in LGG patients. A The fractions of scaffolds, regulators and clients with available gene-expression data in TCGA cohorts. B The expression levels of 3585 LLPS-related genes in LGG tissues from TCGA cohort compared with normal brain tissues from GTEx database. C The distribution of scaffolds, regulators and clients among 225 prognostic LLPS-related DEGs. D-E The top 10 significantly enriched GO terms and KEGG pathways for 225 prognostic LLPS-related DEGs. [file 12967_2022_3266_MOESM1_ESM.tif]

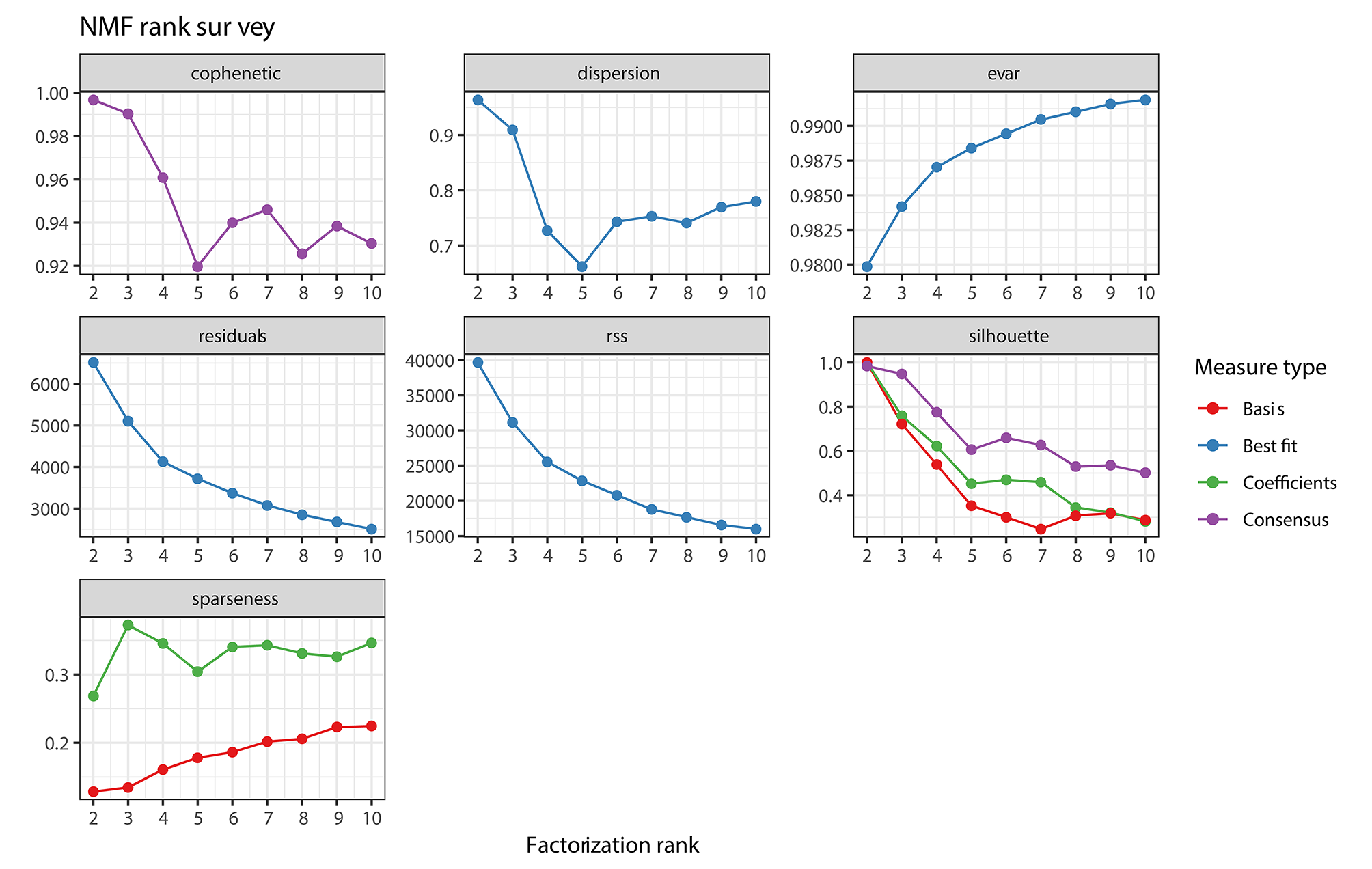

Supplement: Supplementary file 2 — Additional file 2: Fig. S2. The association between cophenetic, dispersion, evar, residuals, rss, silhouette, and sparseness concerning the clustering numbers. [file 12967_2022_3266_MOESM2_ESM.tif]

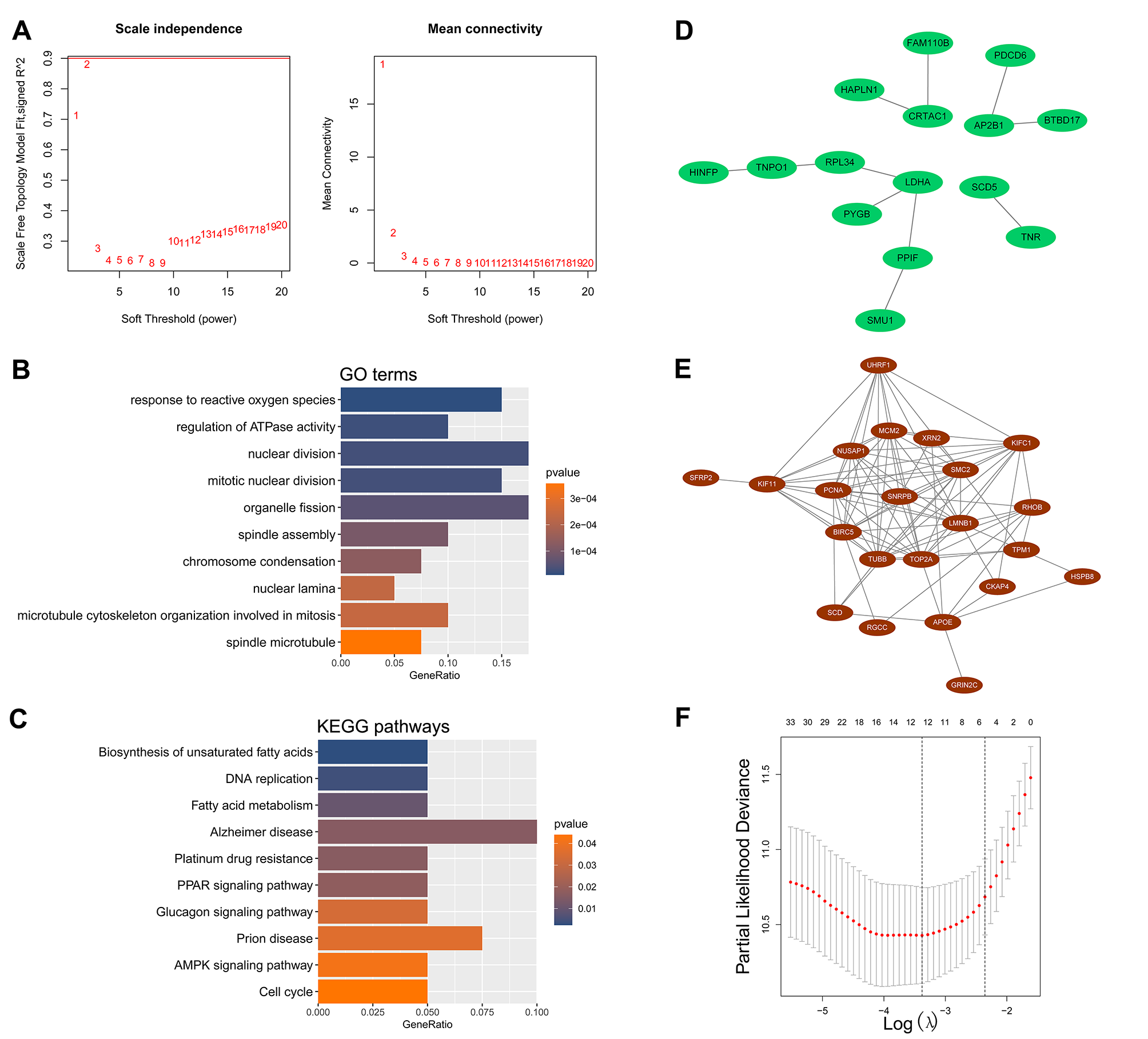

Supplement: Supplementary file 3 — Additional file 3: Fig. S3. Identification of LLPS-related hub genes to construct a a prognostic signature. A The scale independence and mean connectivity plot for selecting soft threshold. B-C The top 10 significantly enriched GO terms and KEGG pathways for the genes in the green and brown modules. D-E The network of the genes in the green and brown modules (weight of edge > 0.15). F Cross-validation for tuning parameter selection in the proportional hazards model. [file 12967_2022_3266_MOESM3_ESM.tif]

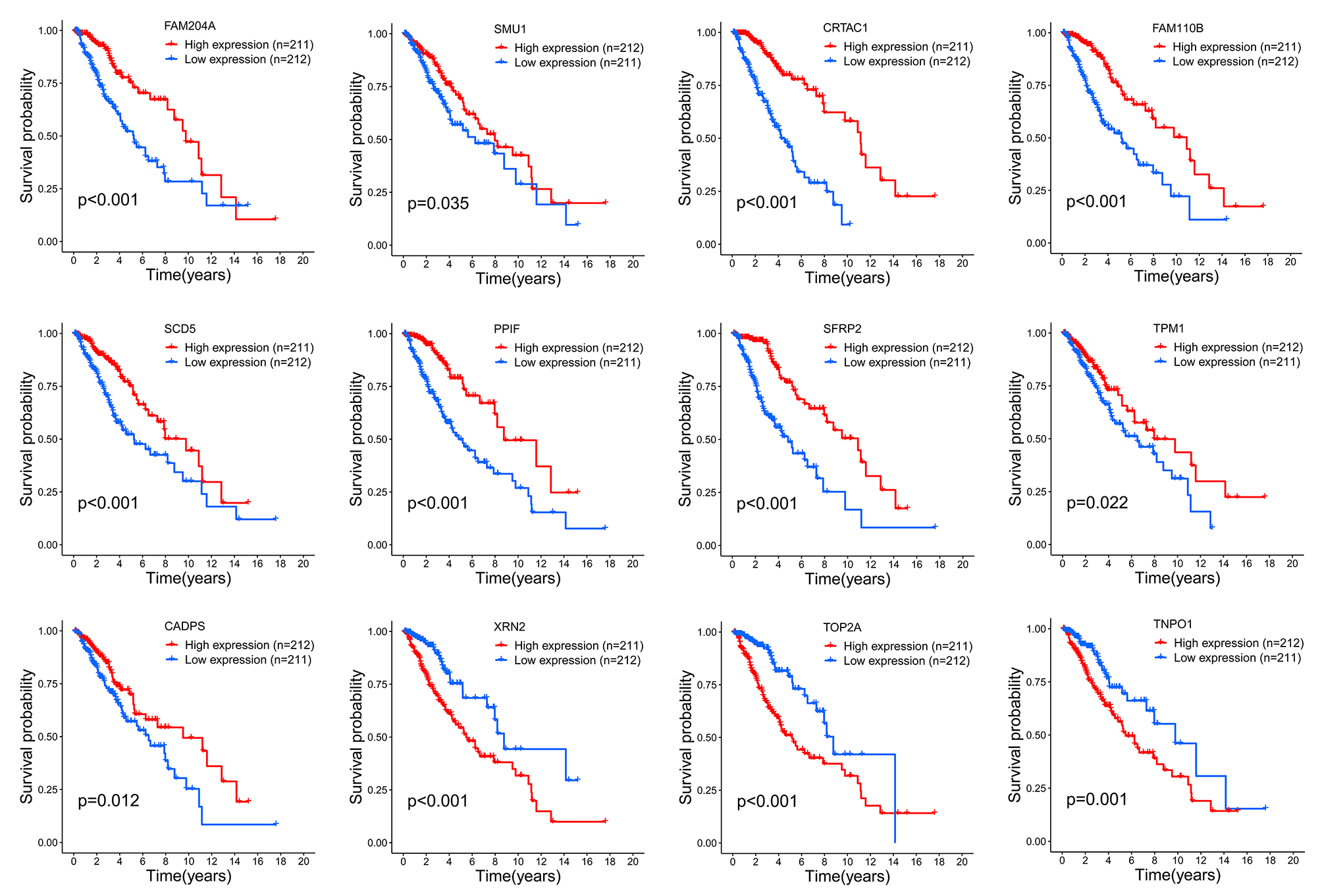

Supplement: Supplementary file 4 — Additional file 4: Fig. S4. The Kaplan–Meier survival curves of 12 selected LLPS-related genes in TCGA cohort. [file 12967_2022_3266_MOESM4_ESM.tif]

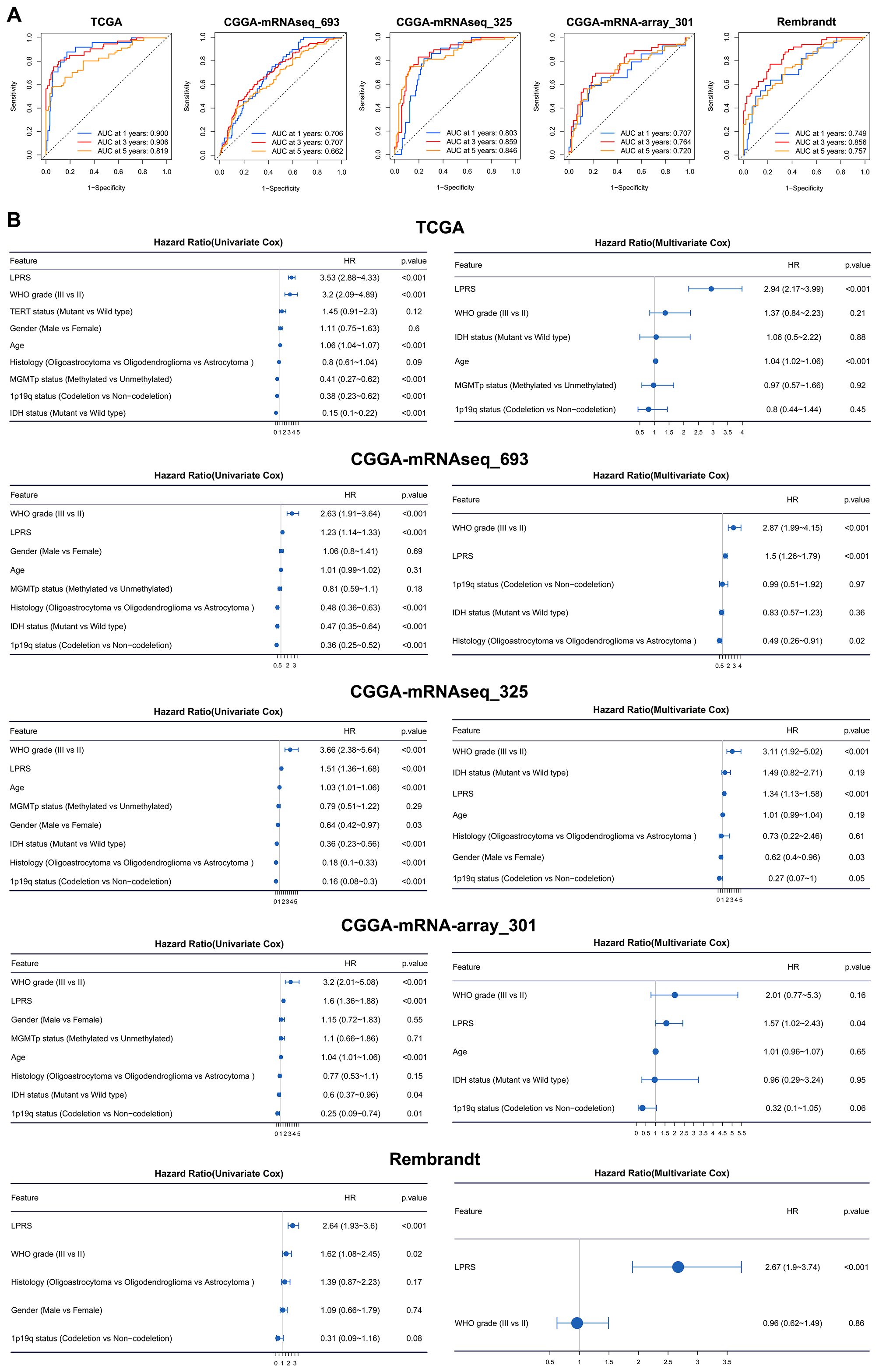

Supplement: Supplementary file 5 — Additional file 5: Fig. S5. The prognostic value of LPRS in multiple cohorts. A ROC curve analyses of LPRS in predicting 1-, 3- and 5-year OS in TCGA, CGGA-mRNAseq_693, CGGA-mRNAseq_325, CGGA-mRNA-arry_301 and Rembrandt cohorts. B The independent prognostic value of LPRS was validated by performing univariate and multivariate Cox regression analyses in TCGA, CGGA-mRNAseq_693, CGGA-mRNAseq_325, CGGA-mRNA-arry_301 and Rembrandt cohorts. [file 12967_2022_3266_MOESM5_ESM.tif]

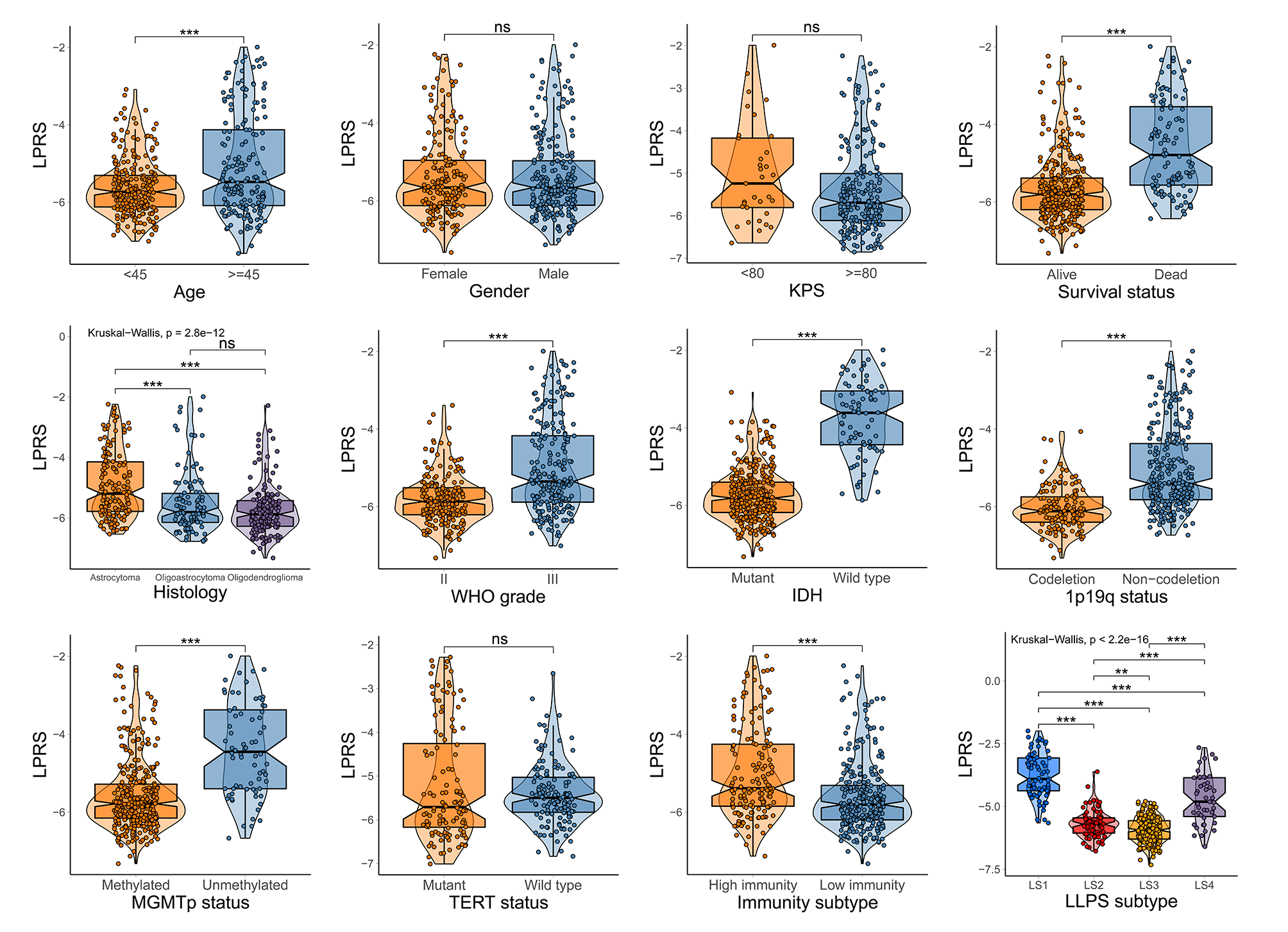

Supplement: Supplementary file 6 — Additional file 6: Fig. S6. The correlations of LPRS with clinicopathological features of LGG patients in TCGA cohort. **P < 0.01, ***P < 0.001, and ns No significance. [file 12967_2022_3266_MOESM6_ESM.tif]

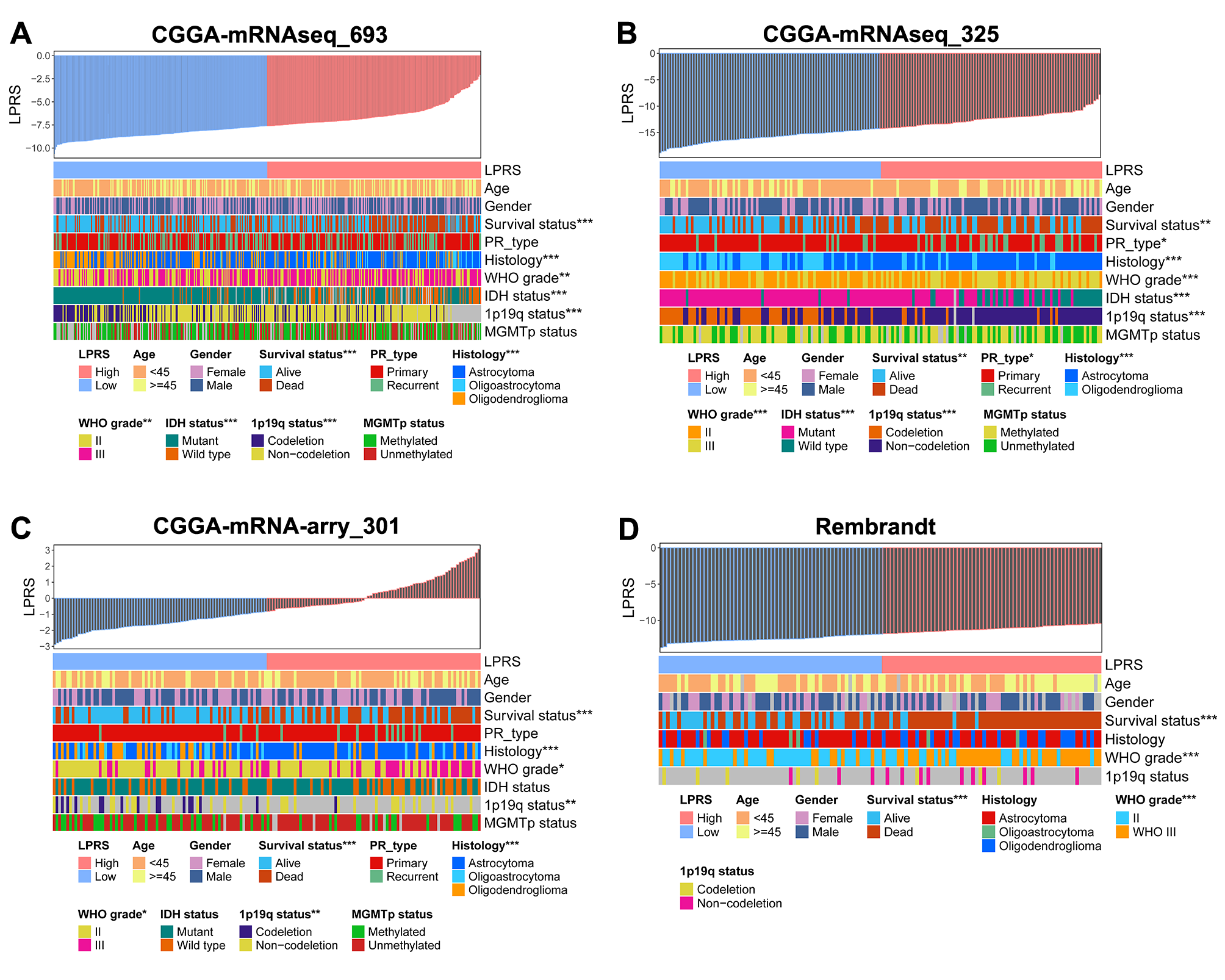

Supplement: Supplementary file 7 — Additional file 7: Fig. S7. Heatmaps showing the correlations of LPRS with clinicopathological features of LGG patients in CGGA-mRNAseq_693 cohort, CGGA-mRNAseq_325 cohort, CGGA-mRNA-arry_301 cohort and Rembrandt cohort. *P < 0.05, **P < 0.01, and ***P < 0.001. [file 12967_2022_3266_MOESM7_ESM.tif]

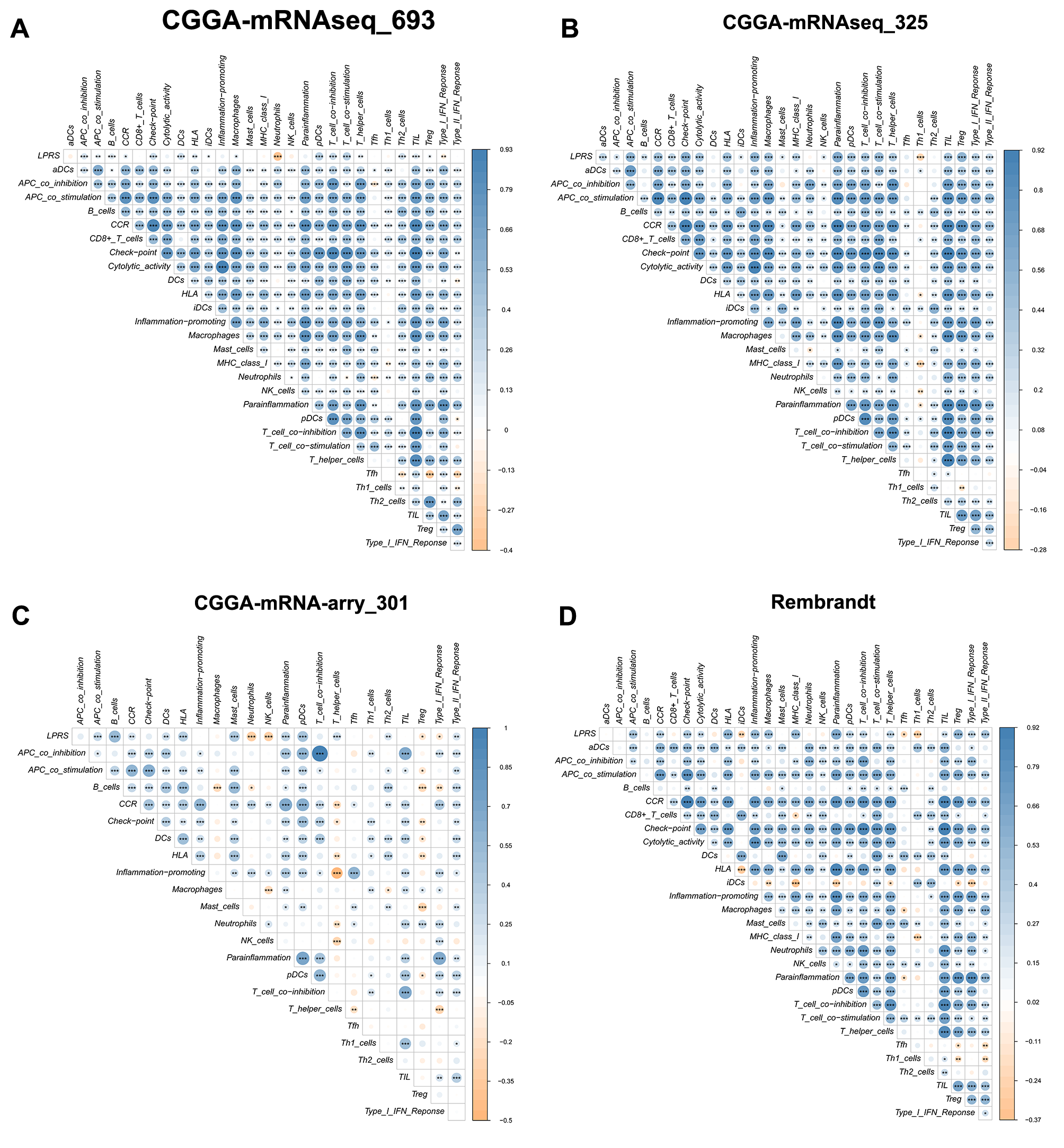

Supplement: Supplementary file 8 — Additional file 8: Fig. S8. Correlations between LPRS and the ssGSEA Z-scores of 29 immune signatures in CGGA-mRNAseq_693 cohort, CGGA-mRNAseq_325 cohort, CGGA-mRNA-arry_301 cohort and Rembrandt cohort. *P < 0.05, **P < 0.01, and ***P < 0.001. [file 12967_2022_3266_MOESM8_ESM.tif]

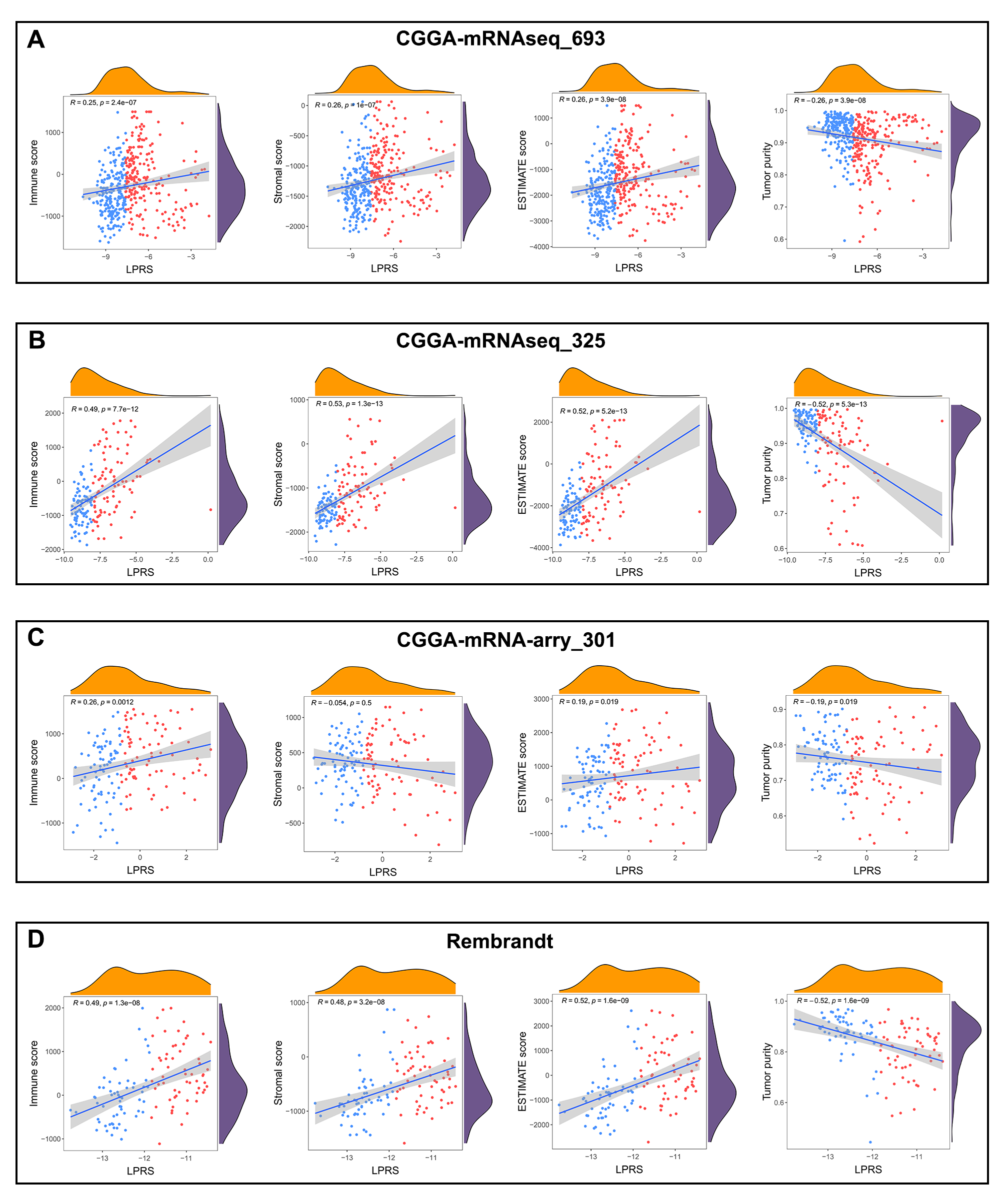

Supplement: Supplementary file 9 — Additional file 9: Fig. S9. Correlations of LPRS with immune scores, stromal scores, ESTIMATE scores and tumor purity in CGGA-mRNAseq_693 cohort, CGGA-mRNAseq_325 cohort, CGGA-mRNA-arry_301 cohort and Rembrandt cohort. [file 12967_2022_3266_MOESM9_ESM.tif]

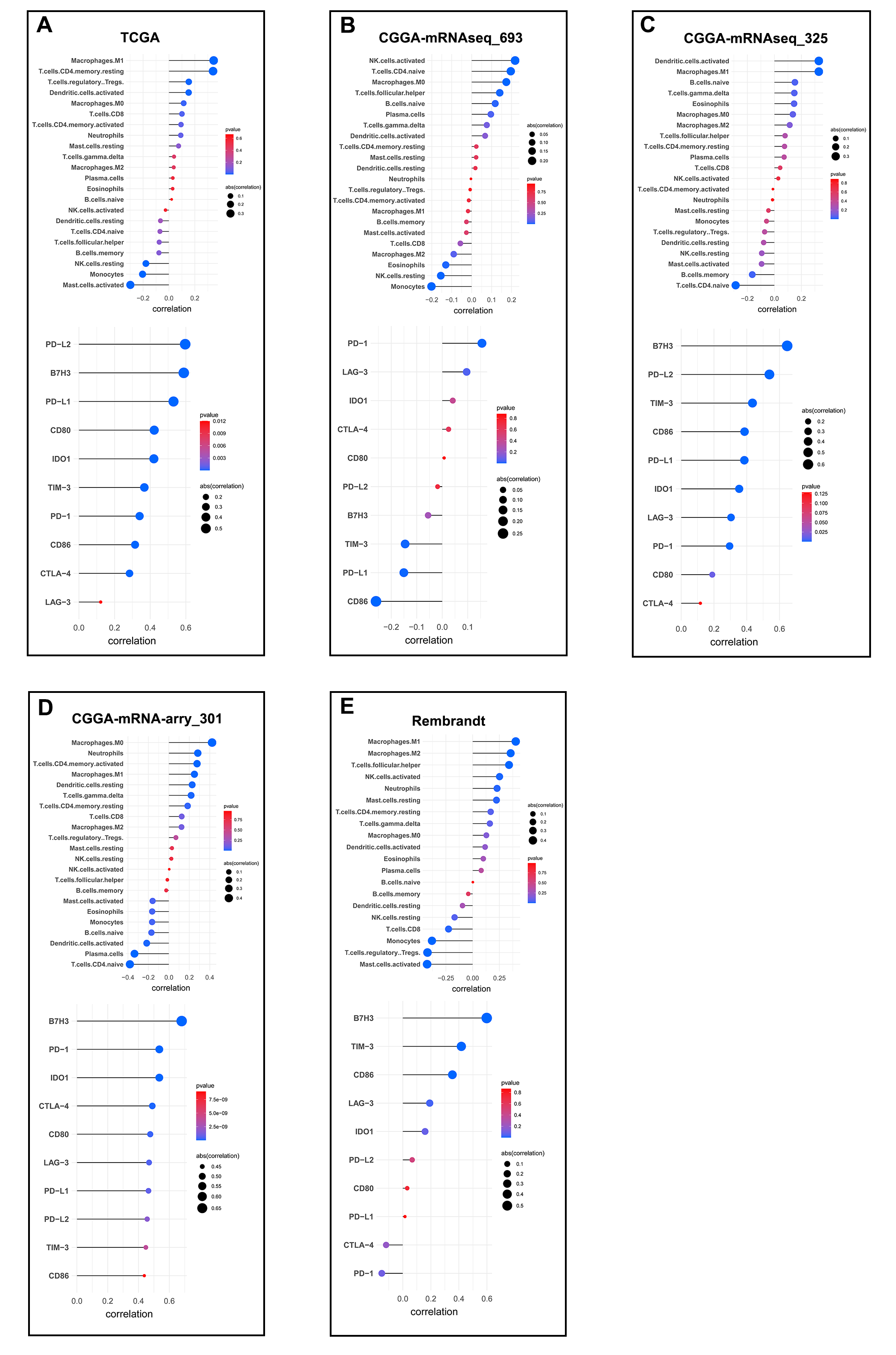

Supplement: Supplementary file 10 — Additional file 10: Fig. S10. Correlations of LPRS with the infiltration levels of 22 immune cells and the expression levels of immune checkpoints in TCGA cohort, CGGA-mRNAseq_693 cohort, CGGA-mRNAseq_325 cohort, CGGA-mRNA-arry_301 cohort and Rembrandt cohort. [file 12967_2022_3266_MOESM10_ESM.tif]
