# Supplementary material for: An integrative multi-omics analysis based on liquid–liquid phase separation delineates distinct subtypes of lower-grade glioma and identifies a prognostic signature
Source: J Transl Med. 2022 Jan 29;20:55. doi: 10.1186/s12967-022-03266-1 (PMC8800244; doi:10.1186/s12967-022-03266-1)
Supplement: Supplementary file 11 — Additional file 11: Table S1. Clinical features of tissues used for qRT-PCR and IHC. [file 12967_2022_3266_MOESM11_ESM.docx]

**Supplementary Table S1. Clinical features of tissues used for qRT-PCR and IHC.**

| **Acute brain injury patients** | | | | | | | | | |
| --- | --- | --- | --- | --- | --- | --- | --- | --- | --- |
| **NO.** | **Gender** | | | **Age** | **qRT-PCR** | | | | **IHC** |
| 1 | Male | | | 36 | YES | | | | NO |
| 2 | Male | | | 54 | YES | | | | YES |
| 3 | Male | | | 63 | YES | | | | YES |
| 4 | Male | | | 75 | YES | | | | NO |
| 5 | Male | | | 65 | YES | | | | YES |
| 6 | Male | | | 58 | YES | | | | YES |
| 7 | Female | | | 71 | YES | | | | YES |
| 8 | Male | | | 58 | YES | | | | YES |
| 9 | Male | | | 65 | YES | | | | YES |
| 10 | Male | | | 65 | YES | | | | YES |
| **Low-grade glioma patients** | | | | | | | | | |
| **NO.** | | **Gender** | **Age** | | | **WHO grade** | **qRT-PCR** | **IHC** | |
| 1 | | Male | 50 | | | II | YES | YES | |
| 2 | | Female | 49 | | | II | YES | YES | |
| 3 | | Male | 36 | | | II | YES | NO | |
| 4 | | Female | 65 | | | II | YES | NO | |
| 5 | | Male | 23 | | | II | YES | NO | |
| 6 | | Female | 62 | | | II | YES | YES | |
| 7 | | Female | 67 | | | II | YES | YES | |
| 8 | | Male | 58 | | | III | YES | YES | |
| 9 | | Female | 49 | | | III | YES | YES | |
| 10 | | Female | 70 | | | III | YES | NO | |
| 11 | | Female | 47 | | | III | YES | YES | |
| 12 | | Female | 38 | | | III | YES | NO | |
| 13 | | Male | 64 | | | III | YES | NO | |
| 14 | | Male | 63 | | | III | YES | YES | |
| 15 | | Female | 37 | | | III | YES | NO | |
