# Supplementary material for: An integrative multi-omics analysis based on liquid–liquid phase separation delineates distinct subtypes of lower-grade glioma and identifies a prognostic signature
Source: J Transl Med. 2022 Jan 29;20:55. doi: 10.1186/s12967-022-03266-1 (PMC8800244; doi:10.1186/s12967-022-03266-1)
Supplement: Supplementary file 12 — Additional file 12: Table S2. The primers sequence used for qRT-PCR. [file 12967_2022_3266_MOESM12_ESM.docx]

**Supplementary Table S2. The primers sequence used in this study.**

| **Name** | **Forward-primer** | **Reverse-primer** |
| --- | --- | --- |
| FAM204A | 5’-CCCAGTGGAAAGAGCTTACTC-3’ | 5’- TCCACAGCCTGGTCTATCCT -3’ |
| SMU1 | 5’-CAGGGGCAGATTGTCAGAAG -3’ | 5’- CAGTAGATCCATTCACCACGG-3’ |
| TNPO1 | 5’-TGCTG GACTCTTAGCCGCTAT-3’ | 5’- CTTGTTGCTGTCCAGGATGC-3’ |
| TOP2A | 5’-GATGACAA CCAGCGTGTTGAG-3’ | 5’- CCACCCAGTACCGATTCCTT-3’ |
| β-actin | 5’-TGACGTGGACATCCGCAAAG-3’ | 5’- CTGGAAGGTGGACAGCGAGG-3’ |
