# Supplementary material for: An integrative multi-omics analysis based on liquid–liquid phase separation delineates distinct subtypes of lower-grade glioma and identifies a prognostic signature
Source: J Transl Med. 2022 Jan 29;20:55. doi: 10.1186/s12967-022-03266-1 (PMC8800244; doi:10.1186/s12967-022-03266-1)
Supplement: Supplementary file 13 — Additional file 13: Table S3. The primary antibodies used in immunohistochemistry (IHC). [file 12967_2022_3266_MOESM13_ESM.docx]

**Supplementary Table S3. The primary antibodies used in immunohistochemistry (IHC).**

| **Protein** | **Antibody Name** | **Antibody Number** | **Company** | **Antibody Concentration** |
| --- | --- | --- | --- | --- |
| FAM204A | FAM204A Rabbit pAb | HPA038182 | Sigma-Aldrich | 1:200 |
| SMU1 | SMU1 Rabbit pAb | 15511-1-AP | Proteintech Group | 1:100 |
| TNPO1 | TNPO1 Rabbit pAb | PAA272HU01 | Cloud-Clone Corp | 1:100 |
| TOP2A | TOP2A Rabbit pAb | A0726 | Abclonal Technology | 1:200 |
