# Supplementary material for: An integrative multi-omics analysis based on liquid–liquid phase separation delineates distinct subtypes of lower-grade glioma and identifies a prognostic signature
Source: J Transl Med. 2022 Jan 29;20:55. doi: 10.1186/s12967-022-03266-1 (PMC8800244; doi:10.1186/s12967-022-03266-1)
Supplement: Supplementary file 14 — Additional file 14: Table S4. The details about the versions and arguments/parameters of important 'R' packages in this work. [file 12967_2022_3266_MOESM14_ESM.docx]

**Table S4.** The details about the versions and arguments/parameters of important 'R' packages in this work.

| **'R' package** | **version** | **function** | **arguments/parameters** |
| --- | --- | --- | --- |
| **clusterProfiler** | 3.18.1 | EnrichKEGG() | organism= "hsa"; pvalueCutoff =1; qvalueCutoff =1; The other arguments/parameters are default values. |
|  |  | EnrichGO() | OrgDb=org.Hs.eg.db; pvalueCutoff =1; qvalueCutoff = 1; ont="all"; readable =T; The other arguments/parameters are default values. |
|  |  |  |  |
| **NMF** | 0.23.0 | nmf() | nrun=50; method = "brunet"; set a seed; The other arguments/parameters are default values. |
|  |  |  |  |
| **Maftools** | 2.6.05 | oncoplot() | top = 30; The other arguments/parameters are default values. |
|  |  |  |  |
| **WGCNA** | 1.70-3 | goodSamplesGenes() | verbose = 3; The other arguments/parameters are default values. |
|  |  | cutreeStatic() | cutHeight = 20000; minSize = 10; The other arguments/parameters are default values. |
|  |  | mergeCloseModules() | cutHeight = 0.25; verbose = 3; The other arguments/parameters are default values. |
|  |  | plotDendroAndColors() | addGuide = TRUE; guideHang = 0.05; |
|  |  |  |  |
| **glmnet** | 1.06 | LASSO | maxit = 1000 |
